# Newcomb–Benford law and the detection of frauds in international trade

**DOI:** 10.1073/pnas.1806617115

**Published:** 2018-12-10

**Authors:** Andrea Cerioli, Lucio Barabesi, Andrea Cerasa, Mario Menegatti, Domenico Perrotta

**Affiliations:** ^a^Department of Economics and Management, University of Parma, 43125 Parma, Italy;; ^b^Department of Economics and Statistics, University of Siena, 53100 Siena, Italy;; ^c^European Commission, Joint Research Centre, 21027 Ispra, Italy

**Keywords:** statistical antifraud analysis, Newcomb–Benford law, customs fraud, customs valuation, anomaly detection

## Abstract

The contrast of fraud in international trade is a crucial task of modern economic regulations. We develop statistical tools for the detection of frauds in customs declarations that rely on the Newcomb–Benford law for significant digits. Our first contribution is to show the features, in the context of a European Union market, of the traders for which the law should hold in the absence of fraudulent data manipulation. Our results shed light on a relevant and debated question, since no general known theory can exactly predict validity of the law for genuine empirical data. We also provide approximations to the distribution of test statistics when the Newcomb–Benford law does not hold. These approximations open the door to the development of modified goodness-of-fit procedures with wide applicability and good inferential properties.

## Significance

The detection of frauds is one of the most prominent applications of the Newcomb–Benford law for significant digits. However, no general theory can exactly anticipate whether this law provides a valid model for genuine, that is, nonfraudulent, empirical observations, whose generating process cannot be known with certainty. Our first aim is then to establish conditions for the validity of the Newcomb–Benford law in the field of international trade data, where frauds typically involve huge amounts of money and constitute a major threat for national budgets. We also provide approximations to the distribution of test statistics when the Newcomb–Benford law does not hold, thus opening the door to the development of statistical procedures with good inferential properties and wide applicability.

The contrast of fraud in international trade, and the corresponding protection of national budgets, is a crucial task of modern economic regulations. To give an idea of the volumes involved, in 2016 the customs duties flowing into the European Union (EU) budget amounted to more than 20 billion euros and provided about 15% of the total own resources of the EU. Huge losses thus occur when the value of imported goods is underreported (e.g., ref. 1). Most statistical antifraud techniques for international transactions fall in the class of unsupervised methods, with outlier detection and (robust) cluster analysis playing a prominent role (2–5). The rationale is that the bulk of international trade data are made of legitimate transactions and major frauds may stand out as highly suspicious anomalies. Considerable emphasis is also put on procedures that provide stringent control of the number of false positives (6), since substantial investigations like the one reported in ref. 1 are demanding and time consuming. A related crucial requirement is the ability to deal with massive datasets of traders and to provide—as automatically as possible—a ranking of their degree of anomaly. This information is essential for the design of efficient and effective audit plans, a major task for customs offices.

In this work we consider fraud detection through the Newcomb–Benford law (NBL). This law defines a probability distribution for patterns of significant digits in real positive numbers. It relies on the intriguing fact that in many natural and human phenomena the leading—that is, the first significant—digits are not uniformly scattered, as one could naively expect, but follow a logarithmic-type distribution. We refer to refs. 7–10 for an historical summary of the NBL, an extensive review of its challenging mathematical properties, and a survey of its more relevant applications.

Despite its long history, the mathematical and statistical challenges of the NBL have been recognized only recently. From a mathematical perspective, appropriate versions of the law appear in integer sequences, such as the celebrated Fibonacci sequence (8) or the factorial sequence (11). The law also emerges in the context of floating-point arithmetic (12), while a deep probabilistic study was carried out by Hill (13). A seminal note by Varian (14) suggested the idea that agreement with the NBL could validate the “reasonableness” of data. Since then, it is now rather well known—mainly due to the work of Nigrini (see ref. 7, for a review of such studies)—that the NBL can be used as a forensic accounting and auditing tool for financial data. The law has been shown to be a valuable starting point for forensic accountants and to be applicable in a number of auditing contexts, such as external, internal, and governmental auditing. It has also been found successful for identifying the presence of misconduct in other domains, including the identification of irregularities in electoral data (15, 16), campaign finance (17), and economic data (18).

Although the cited advances may suggest applicability of the NBL to international trade, there remain major unanswered questions that we address in our work. The first one concerns the trustworthiness of the NBL for genuine—that is, nonfraudulent—transactions. As shown in ref. 19, no general known theory can exactly predict whether the NBL should hold in any specific application, whose data-generating process cannot be known with certainty, even in the absence of fraud or other data manipulations; see also refs. 20–22 for related concerns. Our first goal is then to provide insight on the suitability of the NBL for modeling the distribution of digits of genuine transaction values arising in international trade. We use the Italian import market as a specimen for our study, but our approach is general and can be replicated for any country for which detailed customs data are available. Knowledge of the conditions under which the NBL should be expected to hold in the absence of data manipulation is an essential ingredient for the implementation of large-scale monitoring processes in which tens (or even hundreds) of thousands of traders are screened in an automatic and fast way with the aim of identifying the most suspicious cases. In *SI Appendix*, section 7 we describe a web application that has been developed to assist customs officers and auditors in this screening task, which can be executed in full autonomy on their own datasets. It may instead be very difficult to ascertain whether anomaly should be attributed to fraud or to model failure if the NBL does not provide a suitable model for genuine transactions; see also ref. 23, p. 193, for a similar concern.

Our second goal is to deepen our knowledge of the empirical behavior of NBL-conformance tests by investigating their power under different contamination schemes. The adoption of such tests for antifraud screening is based on the assumption that fabrication of data closely following the law is difficult and that fraudsters might be biased toward simpler digit distributions, such as the discrete uniform or the Dirac. We also quantify the corresponding false positive rates, to make explicit the different and possibly conflicting facets that empirical researchers have to balance in practice.

The third aim of our work is to provide corrections to test statistics when the NBL does not hold. This is typically the case for traders who operate on a limited number of products, so that there is not enough variability in their transactions. Even if the NBL is not a suitable model for genuine transaction digits, the conformance tests based on our modified statistics have the appropriate empirical size in the absence of data manipulation, while the usual tests turn out to be potentially very liberal. We argue that, having the required size under general trade conditions and being competitive in terms of power, the conformance tests based on our modified statistics are recommended. Therefore, they extend the applicability of large-scale monitoring processes of international trade data to a wider range of practical situations.

## The NBL

### Statistical Background.

Let $D_{1}\left(x\right),D_{2}\left(x\right),\ldots ,$ be the first, the second, …, significant digit of the positive real number x. Let X be a positive real random variable defined on the probability space (Ω,F,P). The NBL implies (and vice versa) that the following joint probability function holds for each $k\in Z^{+}$,

$$\begin{array}{ccc} \rho_{k}\left(d_{1},\ldots ,d_{k}\right) & = & P\left(D_{1}\left(X\right)=d_{1},\ldots ,D_{k}\left(X\right)=d_{k}\right)\\ & = & \log_{10}\left(1+\frac{1}{\sum_{l=1}^{k}10^{k-l}d_{l}}\right), \end{array}$$ [1]

where $d_{1}\in \left\{1,\ldots ,9\right\}$ and $d_{l}\in \left\{0,\ldots ,9\right\}$ for l=2,…,k. A practically important special case is that of the first two significant digits (k=2), for which Eq. **1** reduces to

$$\rho_{2}\left(d_{1},d_{2}\right)=\log_{10}\left(1+\frac{1}{10d_{1}+d_{2}}\right).$$ [2]

Similarly, the marginal probability function of $D_{1}\left(X\right)$ is

$$P\left(D_{1}\left(X\right)=d_{1}\right)=\log_{10}\left(1+\frac{1}{d_{1}}\right),$$

while the marginal probability function of $D_{2}\left(X\right)$ is

$$P\left(D_{2}\left(X\right)=d_{2}\right)=\overset{9}{\underset{d_{1}=1}{\sum }}\log_{10}\left(1+\frac{1}{10d_{1}+d_{2}}\right).$$

We refer to ref. 24 for a summary of the mechanisms that give rise to NBL-distributed data in accounting and finance. Among these, there are several statistical motivations for adopting the NBL as a model for the digits appearing in genuine international transactions. A major methodological basis relies on a limit theorem derived by Hill (13), to which we refer for the technical details. A key mathematical concept is that of a random probability measure, which is a function P:Ω→M—where M is the space of probability measures on R—defined on the underlying probability space (Ω,F,P). For each Borel set B the function ω↦P(ω)(B) is a random variable; that is, P(ω) is a probability measure on R for each ω∈Ω. Another important related concept is that of a sequence of P-random M samples, where $M\in Z^{+}$. It is a sequence ${\left(X_{n}\right)}_{n\geq 1}$ of random variables defined on (Ω,F,P) such that, for each ω∈Ω, the first M random variables are drawn independently from the same random probability distribution $P_{1}\left(\omega \right)$, selected according to the random probability measure P, the M subsequent random variables are drawn independently from the same random probability distribution $P_{2}\left(\omega \right)$, in turn selected according to the random probability measure P, and so on. Hill’s limit theorem then states that, if P satisfies some invariance conditions related to either the scale or the base of measurement, for each $M\in Z^{+}$ the P-random M-samples sequence ${\left(X_{n}\right)}_{n\geq 1}$ converges to the NBL with probability one. That is, for each $k\in Z^{+}$ and for i=1,…,n, as n→∞

$$\begin{array}{ccc} \frac{card\left\{i:D_{1}\left(X_{i}\right)=d_{1},\ldots ,D_{k}\left(X_{i}\right)=d_{k}\right\}}{n}\overset{a.s.}{\to }\rho_{k}\left(d_{1},\ldots ,d_{k}\right). & & \end{array}$$ [3]

A second reason for adopting the NBL is that multiplicative processes—which are at the heart of many financial data—generate NBL-distributed data. More precisely, if ${\left(X_{n}\right)}_{n\geq 1}$ is a sequence of independent and identically distributed random variables such that $P\left(X_{1}=0\right)=0$, as n→∞ the sequence ${\left(\prod_{i=1}^{n}X_{i}\right)}_{n\geq 1}$ converges to the NBL with probability one (theorem 8.16 in ref. 8). It can be shown that convergence is extremely fast since it is exponential in n (25). It is also remarkable that, given two independent random variables X and Y only one of which follows the NBL, the product XY is distributed according the NBL provided that P(XY=0)=0 (theorem 8.12 in ref. 8). Finally, NBL-distributed data may also originate from random variables raised to integer powers. If X is an absolutely continuous random variable, as n→∞ the sequence ${\left(X^{n}\right)}_{n\geq 1}$ converges to the NBL with probability one (theorem 8.8 in ref. 8).

### Relevance for International Trade.

Our applied focus is on transactions involving EU traders; we refer to *SI Appendix*, sections 3 and 7 for the institutional regulations supporting their analysis. By international trade data we mean the data collected by EU member states for imports and exports that are declared by national traders and shipping agents using the form called the Single Administrative Document (SAD). The value that we analyze for antifraud purposes is the “statistical value” reported in each SAD, which also includes the costs of insurance and freight (CIF) and is given in euros by taking into account the exchange rate (26). Our interest is then on random variables $X_{1},\ldots ,X_{n}$ defined on the product space

$$X_{i}=U_{i}Q_{i},\;i=1,\ldots ,n,$$ [4]

where $U_{i}$ and $Q_{i}$ are nonnegative random variables representing the (CIF-type) unit price in euros and the traded quantity in transaction i. If we rephrase **[3]** in the context of trade, n corresponds to the number of transactions made by the trader of interest, so that $X_{1},\ldots ,X_{n}$ is the available sample of transaction values, and the ratio m=n/M is the corresponding number of traded goods (provided that m is an integer).

There are different economic reasons suggesting that the distribution of the significant digits contained in $X_{1},\ldots ,X_{n}$ may, under some conditions, be well approximated by the NBL. First, markets are hit by specific shocks and show peculiar reactions to common shocks (27). This, coupled with differences in the trader size and product quality, generates different economic processes for prices and quantities determination, which imply in turn that the observed data of prices and quantities may be described by different trader-specific probability distributions, not exactly predictable in advance. In view of **[3]**, it is then sensible to anticipate good conformance to the NBL when a trader operates by importing or exporting a sufficiently large number of different goods, even if none of the product-specific marginal distributions of digits follows the law. The economic literature also shows that traders have different degrees of market power. Trading operations are affected by market and country features, such as different trade costs and different access to credit (e.g., ref. 28). Therefore, transactions made with different counterparties may be characterized by different economic processes, yielding distributions for transaction values that can be conceived to vary randomly from one product to another for each trader. The significant-digit distribution in international transactions can thus be expected to adhere to the NBL when the trader makes a sufficiently large number of operations, with a sufficiently large number of counterparties, possibly located in different countries.

## A Contamination Model for Fraud

### The Model.

We phrase our antifraud approach within the framework of a trader-specific contamination model where each fraud corresponds to an outlier. For this purpose, we need a slight change in notation and we write $n_{t}$ for the number of transactions made by trader t, which operates on $m_{t}$ distinct products and for which the positive random variable $X^{\left(t\right)}$ now represents a transaction value. We then define

$$\pi_{k}^{\left(t\right)}\left(d_{1},\ldots ,d_{k}\right)=P\left(D_{1}\left(X^{\left(t\right)}\right)=d_{1},\ldots ,D_{k}\left(X^{\left(t\right)}\right)=d_{k}\right),$$

and let T denote the total number of traders in the market.

For t=1,…,T and each $k\in Z^{+}$, the general form of our contamination model is

$$\begin{array}{ccc} \pi_{k}^{\left(t\right)}\left(d_{1},\ldots ,d_{k}\right)=\left(1-\tau_{t}\right)\Psi_{k}^{\left(t\right)}\left(d_{1},\ldots ,d_{k}\right)+\tau_{t}{ϒ}_{k}^{\left(t\right)}\left(d_{1},\ldots ,d_{k}\right), & & \end{array}$$ [5]

where $\Psi_{k}^{\left(t\right)}\left(d_{1},\ldots ,d_{k}\right)$ is the probability of observing $\left\{D_{1}\left(X^{\left(t\right)}\right)=d_{1},\ldots ,D_{k}\left(X^{\left(t\right)}\right)=d_{k}\right\}$ in the absence of fraud, ${ϒ}_{k}^{\left(t\right)}\left(d_{1},\ldots ,d_{k}\right)$ is the probability of the same event for a manipulated transaction, and $0\leq \tau_{t}\leq 1$ is the probability of fraud for trader t. Although it is convenient to work in the digit space through $\pi_{k}^{\left(t\right)}\left(d_{1},\ldots ,d_{k}\right)$, model **5** has a counterpart in the transaction space defined by $X^{\left(t\right)}$. The latter is given in *SI Appendix*, section 1.

Model **5** provides a principled framework for antifraud analysis of international trade data. Indeed, trader t may be considered a potential fraudster if the null hypothesis

$$H_{0}^{\left(t\right)}:\tau_{t}=0$$ [6]

is rejected, in favor of the alternative $H_{1}^{\left(t\right)}:\tau_{t}>0$, based on $n_{t}$ independent copies of $X^{\left(t\right)}$, say $X_{1}^{\left(t\right)},\ldots ,X_{n_{t}}^{\left(t\right)}$.

A useful tractable version of contamination model **5** assumes that the probability of observing a given k-ple of digits in a genuine transaction of trader t depends on the trader features only through the values of $m_{t}$ and $n_{t}$; that is,

$$\Psi_{k}^{\left(t\right)}\left(d_{1},\ldots ,d_{k}\right)≔\;\Psi_{k}^{\left(m_{t},n_{t}\right)}\left(d_{1},\ldots ,d_{k}\right).$$

Therefore, for each $k\in Z^{+}$, the model becomes

$$\begin{array}{ccc} \pi_{k}^{\left(t\right)}\left(d_{1},\ldots ,d_{k}\right) & = & \left(1-\tau_{t}\right)\Psi_{k}^{\left(m_{t},n_{t}\right)}\left(d_{1},\ldots ,d_{k}\right)\\ & & +\;\tau_{t}{ϒ}_{k}^{\left(t\right)}\left(d_{1},\ldots ,d_{k}\right), \end{array}$$ [7]

with **[6]** again stating the absence of fraud. Model **7** implies that the random vector $\left(D_{1}\left(X^{\left(t\right)}\right),\ldots ,D_{k}\left(X^{\left(t\right)}\right)\right)$ is independent of any other trader-specific random variable, given the values of $m_{t}$ and $n_{t}$. Although this structure is clearly an approximation, it is coherent with the discussion about the economic elements that make the NBL a plausible model for the digit distribution in genuine international transactions.

A further bonus of models **5** and **7** is that they make clear the antifraud advantages of our methodology over the often uninformative analysis of aggregated data, as given, for example, in ref. 18. In the latter instance, for each $k\in Z^{+}$, the underlying contamination model would be

$$\pi_{k}\left(d_{1},\ldots ,d_{k}\right)=\left(1-\tau \right)\Psi_{k}\left(d_{1},\ldots ,d_{k}\right)+\tau {ϒ}_{k}\left(d_{1},\ldots ,d_{k}\right),$$

where the quantities involved are now constant for the whole (product-specific) market. Testing the hypothesis that τ=0 in this restricted model requires a sample $X_{1},\ldots ,X_{T}$ obtained from T traders, for which just one replicate is available. However, the inferential conclusion that τ>0 is much less informative than rejection of **[6]** for some t∈{1,…,T}. In fact, τ>0 yields no information on the specific traders that are responsible for rejection and identification of the fraudsters must be left to further nonstatistical investigations. Another notable advantage is that models **5** and **7** acknowledge the existence of a trader-specific propensity to fraud.

### Testing the Absence of Fraud.

The usual hypothesis of interest in the antifraud literature (7, 10) is

$$H_{0}^{\left(t\right)}:\pi_{k}^{\left(t\right)}\left(d_{1},\ldots ,d_{k}\right)=\rho_{k}\left(d_{1},\ldots ,d_{k}\right),\;\forall \;k\in Z^{+},$$ [8]

which corresponds to **[6]** when $\Psi_{k}^{\left(t\right)}\left(d_{1},\ldots ,d_{k}\right)$ is the NBL. Several statistics exist for testing **[8]** for a given value of k, the simplest one being the χ^2^ statistic

$$\begin{array}{ccc} V_{\left\{1,\ldots ,k\right\}}^{\left(t\right)}=\underset{d_{1},\ldots ,d_{k}}{\sum }\frac{{\left(N_{k}^{\left(t\right)}\left(d_{1},\ldots ,d_{k}\right)-n_{t}\rho_{k}\left(d_{1},\ldots ,d_{k}\right)\right)}^{2}}{n_{t}\rho_{k}\left(d_{1},\ldots ,d_{k}\right)}, & & \end{array}$$ [9]

where $N_{k}^{\left(t\right)}\left(d_{1},\ldots ,d_{k}\right)$ is the frequency of the k-ple $\left(d_{1},\ldots ,d_{k}\right)$ in the sample of $n_{t}$ transactions for trader t. It is a standard result that, as $n_{t}\to \infty$, $V_{\left\{1,\ldots ,k\right\}}^{\left(t\right)}\overset{L}{\to }\chi_{\nu }^{2}$ when **[8]** is true, with $\nu =9\times 10^{k-1}-1$. In practice only NBL marginals of low order are analyzed. The two-digit version of **[9]**, that is, $V_{\left\{1,2\right\}}^{\left(t\right)}$, tests the fit to the 2D marginal of the NBL given in **[2]**, while the corresponding 1D marginal hypotheses are tested through $V_{\left\{1\right\}}^{\left(t\right)}$ and $V_{\left\{2\right\}}^{\left(t\right)}$, respectively.

In our empirical study we also consider the multiple-stage approach proposed by Barabesi et al. (6) with the aim of introducing a more stringent control on the proportion of false discoveries. This approach tests a decreasing sequence of lower-dimensional marginals of the NBL through their exact conditional distributions. Specifically, in the simple two-step version that we consider here, the method of Barabesi et al. (6) first tests the two-digit marginal **2** of the NBL by comparing $V_{\left\{1,2\right\}}^{\left(t\right)}$ to the quantiles of its exact distribution under the null, which are approximated through an efficient Monte Carlo scheme. Then, if the 2D NBL is rejected, the fit to the 1D marginals is tested by $V_{\left\{1\right\}}^{\left(t\right)}$ and $V_{\left\{2\right\}}^{\left(t\right)}$. These lower-dimensional tests use the exact conditional distributions of $V_{\left\{1\right\}}^{\left(t\right)}$ and $V_{\left\{2\right\}}^{\left(t\right)}$, given rejection of the 2D hypothesis, instead of their marginal ones. Type-I error rates are thus controlled at the prescribed level (e.g., 1%) at each step of the procedure, both in the two-digit and in the one-digit tests. Furthermore, the outcome on the one-digit tests reveals which digit is responsible for nonconformance to **[2]**.

Since χ^2^ tests may also have some shortcomings (ref. 10, chap. 37), additional procedures not based on **[9]** and less formal methods are considered in *SI Appendix*, sections 5 and 6. Qualitative findings are similar in all cases. Nevertheless, for our purposes it is instructive to look at the results for χ^2^ tests, because their distribution (either exact or asymptotic) is known under the NBL. We can thus look at the agreement between the empirical and the nominal distribution of the test statistics to assess whether genuine transactions actually follow the law, that is, if $\Psi_{k}^{\left(t\right)}\left(d_{1},\ldots ,d_{k}\right)$ in **[5]** (or $\Psi_{k}^{\left(m_{t},n_{t}\right)}\left(d_{1},\ldots ,d_{k}\right)$ in **[7]**) is the NBL.

## Adequacy of the NBL for Trade Data

Although the theoretical results sketched in the statistical background and the subsequent economic arguments broadly motivate the adoption of the NBL as a sensible model for genuine transactions in the context of international trade, it is unclear how they may fit to empirical transactions whose generating mechanism cannot be exactly known and obviously involves only a finite number of terms. One goal of our study is then to provide evidence on the quality of the NBL assumption **1** to the digit distribution of transaction values for noncheating traders that operate in real international markets. For this purpose, we assume that our contamination model holds with $\tau_{t}=0$ for each trader. We also take **[7]** as a sensible and practically workable approximation to this model in the absence of a priori information on the trader.

We simulate nonmanipulated statistical values, according to definition **4**, for $T^{†}$ “idealized” traders in each relevant configuration of trade represented by a pair $\left(m_{t},n_{t}\right)$. For this aim, we sample transactions with replacement from the Cartesian product spaces

$$X_{j}=U_{j}\times Q_{j},\;j=1,\ldots ,G,$$ [10]

where $U_{j}=\left\{u_{1},\ldots ,u_{n_{j}}\right\}$ and $Q_{j}=\left\{q_{1},\ldots ,q_{n_{j}}\right\}$ denote the sets of unit prices (CIF-type) and traded quantities, respectively, originated in all of the market transactions involving good j, $n_{j}$ is the number of such transactions, and G is the total number of goods in the market. The details of the simulation algorithm are reported in *SI Appendix*, section 2. In our experimental setting the values of $m_{t}$ and $n_{t}$ are fixed by design, while in empirical analysis we instead condition on the observed values of $m_{t}$ and $n_{t}$ for the trader under scrutiny. We replicate genuine international trading behavior in one specific EU market by picking unit price and traded quantity at random from the database of one calendar year Italian customs declarations, after appropriate trader and product anonymization making it impossible to infer the features of individual operators. Two databases of simulated transactions (pseudo-datasets) similar to those analyzed in this work can be accessed through *SI Appendix*, section 3, where their structure is explained. A description of our code is also given in *SI Appendix*, section 3.

For each idealized trader t and a chosen value of k, we compare the observed distribution of digits to the theoretical NBL values **1** through the test statistic $V_{\left\{1,\ldots ,k\right\}}^{\left(t\right)}$. This statistic will be asymptotically distributed as $\chi_{\nu }^{2}$ if $\Psi_{k}^{\left(m_{t},n_{t}\right)}\left(d_{1},\ldots ,d_{k}\right)$ is indeed the NBL. Furthermore, its exact distribution under the k-digit NBL hypothesis can be approximated to an arbitrary degree of accuracy through the Monte Carlo approach of Barabesi et al. (6). We thus take the discrepancy between the estimated distribution of $V_{\left\{1,\ldots ,k\right\}}^{\left(t\right)}$, computed by averaging over the $T^{†}$ Monte Carlo replicates of t, and its reference null distribution, say $F_{V_{\left\{1,\ldots ,k\right\}}^{\left(t\right)}}$, as a measure of the adequacy of the NBL assumption in model **7**. Formally, let $\zeta_{\gamma }$ be the γ quantile of $F_{V_{\left\{1,\ldots ,k\right\}}^{\left(t\right)}}$ and let $I_{C}$ denote the indicator function of a given set C. Our Monte Carlo estimate is computed as

$$\hat{\alpha }=\frac{1}{T^{†}}\overset{T^{†}}{\underset{t=1}{\sum }}I_{]\zeta_{1-\alpha },+\infty [}\left(V_{\left\{1,\ldots ,k\right\}}^{\left(t\right)}\right),$$ [11]

for α in the usual range of significance levels. Although a value of $\hat{\alpha }$ close to α does not imply that the empirical distribution of $V_{\left\{1,\ldots ,k\right\}}^{\left(t\right)}$ is well approximated by $F_{V_{\left\{1,\ldots ,k\right\}}^{\left(t\right)}}$ over all its support, it tells us that the approximation is satisfactory for the purpose for which $V_{\left\{1,\ldots ,k\right\}}^{\left(t\right)}$ is computed in antifraud analysis. The insight that we gain from our study is twofold. First, we shed light on the trading configurations—represented in terms of pairs $\left(m_{t},n_{t}\right)$—that ensure close agreement between $\Psi_{k}^{\left(m_{t},n_{t}\right)}\left(d_{1},\ldots ,d_{k}\right)$ and the NBL in the market from which all of the sets $U_{j}$ and $Q_{j}$ are obtained. Second, we explore the effect of sparseness of digit counts on the distribution of $V_{\left\{1,\ldots ,k\right\}}^{\left(t\right)}$ when $n_{t}$ is small or moderate.

The bulk of our results deal with the simple first-digit statistic $V_{\left\{1\right\}}^{\left(t\right)}$, which is likely to be method of choice by many antifraud practitioners in automated large-scale auditing processes. As a reference, we also provide the estimated test sizes for the two-stage (TS) version of the procedure of Barabesi et al. (6) and for the two-digit statistic $V_{\left\{1,2\right\}}^{\left(t\right)}$. The former is intended to be a reasonable compromise between simplicity of use and strong reduction in the rate of false detections, while the latter is often recommended in applications with not-too-small sample sizes (ref. 7, p. 79). We estimate test sizes using **[11]** for a wide range of pairs $\left(m_{t},n_{t}\right)$, with $m_{t}\leq n_{t}$. The chosen grid represents the features of some of the most relevant traders in the empirical analysis of customs declarations. In fact, the importers for which $n_{t}<50$ cover less than 14% of the recorded transactions in our customs database and an even smaller quota in terms of traded values. Very big traders are not common: To give an idea, $n_{t}>2,000$ for less than 0.1% of the importers in the database, and almost 40% of the recorded transactions refer to traders with $50\leq n_{t}\leq 2,000$. We present only the findings for the case α=0.01, similar conclusions being valid for other significance levels.

Table 1 displays the estimated sizes of the test of the first-digit marginal hypothesis for both $V_{\left\{1\right\}}^{\left(t\right)}$ (using the quantiles of its asymptotic distribution) and TS. These estimates are computed on $T^{†}=85,500$ idealized noncheating traders, pooled across different scenarios with the same pair $\left(m_{t},n_{t}\right)$. One striking feature of the reported values of $\hat{\alpha }$ in Table 1 is that they vary considerably according to the specific trading configuration. This result clearly supports the conjecture that in a realistic market scenario both $m_{t}$ and $n_{t}$ are crucial factors in determining the adequacy of the NBL as a valid model for the empirical digit distribution in the absence of data manipulation. If only one digit is considered, a sample size of $n_{t}=50$ transactions can be considered sufficiently large to justify the asymptotic $\chi_{8}^{2}$ approximation to the distribution of $V_{\left\{1\right\}}^{\left(t\right)}$ and the adoption of the NBL as a reasonable model for $\Psi_{1}^{\left(m_{t},n_{t}\right)}\left(d_{1}\right)$, provided that the number of traded products is large as well (around 20, say). Similar findings hold for all of the pairs $\left(m_{t},n_{t}\right)$ taken into account in our experiment and provide an empirical verification of the speed of convergence to the NBL anticipated by the asymptotic framework of Hill’s result **3**. An interesting remark is that $\hat{\alpha }$ for $V_{\left\{1\right\}}^{\left(t\right)}$ is closer to α when $m_{t}=n_{t}$, thus suggesting that convergence in **[3]** is faster when M=1. On the other hand, TS yields a very conservative test when the NBL provides a satisfactory model. This result is hardly surprising, since TS tests the first-digit hypothesis at nominal size α in the conditional distribution of $V_{\left\{1\right\}}^{\left(t\right)}$, given previous rejection of the two-digit NBL hypothesis. In *SI Appendix*, section 5, we also investigate the fit of the whole empirical distribution of $V_{\left\{1\right\}}^{\left(t\right)}$ to the nominal $\chi_{8}^{2}$ distribution.

**Table 1.** Estimated test sizes (Eq. 11) for the first-digit statistic $V_{\left\{1\right\}}^{\left(t\right)}$, using the asymptotic quantile $\chi_{8,0.99}^{2}$, and for the TS version of the procedure of Barabesi et al. (6), based on $T^{†}=85,500$ Monte Carlo replicates for each configuration $\left(m_{t},n_{t}\right)$, with $m_{t}\leq n_{t}$

		$m_{t}$								
No. of transactions	Test	1	5	10	20	40	80	100	200	500
$n_{t}=50$	$V_{\left\{1\right\}}^{\left(t\right)}$	0.053	0.027	0.018	0.014	0.011	—	—	—	—
	TS	0.024	0.003	0.001	0.000	0.000	—	—	—	—
$n_{t}=100$	$V_{\left\{1\right\}}^{\left(t\right)}$	0.071	0.045	0.027	0.016	0.012	0.011	0.011	—	—
	TS	0.049	0.013	0.004	0.001	0.000	0.000	0.000	—	—
$n_{t}=200$	$V_{\left\{1\right\}}^{\left(t\right)}$	0.094	0.069	0.047	0.026	0.016	0.012	0.011	0.010	—
	TS	0.070	0.035	0.013	0.003	0.001	0.000	0.000	0.000	—
$n_{t}=500$	$V_{\left\{1\right\}}^{\left(t\right)}$	0.132	0.126	0.097	0.062	0.031	0.017	0.016	0.012	0.010
	TS	0.103	0.084	0.049	0.017	0.003	0.000	0.000	0.000	0.000

Model **7** holds with $\tau_{t}=0$ for each trader. The nominal test size is α=0.01.

Our results point to the conclusion that the NBL is not a satisfactory model when $m_{t}$ is much smaller than $n_{t}$. This statement is verified consistently over all market configurations and does not depend on the specific testing methodology. Indeed, also the potentially very conservative TS procedure can become considerably liberal if $m_{t}\ll n_{t}$. The same is true for other adjustments to $V_{\left\{1\right\}}^{\left(t\right)}$ that control for multiplicity of tests among traders, not reported here. We argue that lack of variability in the transactions made by trader t is the main reason for the discrepancy between the NBL and $\Psi_{k}^{\left(m_{t},n_{t}\right)}\left(d_{1},\ldots ,d_{k}\right)$ when $m_{t}$ is small. Whatever the interpretation, our simulation results confirm that the asymptotic framework set by **[3]** does not hold if $m_{t}=o\left(n_{t}\right)$, requiring instead $m_{t}=O\left(n_{t}\right)$. Our results also quantify how much deleterious can be the effect of keeping $m_{t}$ fixed on the distribution of test statistics. Indeed, they show that in this setting an increase of the sample size $n_{t}$ worsens the situation, since it points to a “wrong” asymptotic direction. The clear message is then that standard conformance tests, such as $V_{\left\{1,\ldots ,k\right\}}^{\left(t\right)}$, should not be used for antifraud purposes when $m_{t}\ll n_{t}$, because the hypotheses **6** and **8** cannot be taken any longer to be equivalent.

We conclude this section with a glimpse of the performance of the two-digit statistic $V_{\left\{1,2\right\}}^{\left(t\right)}$, when either the asymptotic quantile $\chi_{89,0.99}^{2}$ or the exact 0.99 quantile from Barabesi et al. (6) is used. The estimated test sizes, now based on $T^{†}=28,500$ Monte Carlo replicates for each configuration $\left(m_{t},n_{t}\right)$, are reported in Table 2. As expected, convergence to the $\chi_{89}^{2}$ distribution is slower than convergence to $\chi_{8}^{2}$ in the one-digit case. The adoption of exact quantiles should thus be preferred with $V_{\left\{1,2\right\}}^{\left(t\right)}$, except in the instance of large values of both $n_{t}$ and $m_{t}$. Our results confirm the relationship between accuracy of the NBL approximation and the ratio $m_{t}/n_{t}$, suggesting $m_{t}\geq 0.2n_{t}$ as a sensible rule of thumb when the exact quantiles are used. They also provide a clue of the strategy to be adopted with more complex large-k procedures.

**Table 2.** Estimated test sizes (Eq. 11) for the two-digit statistic $V_{\left\{1,2\right\}}^{\left(t\right)}$, using the asymptotic quantile $\chi_{89,0.99}^{2}$ (As) and the exact 0.99 quantile (Ex) from Barabesi et al. (6), based on $T^{†}=28,500$ Monte Carlo replicates for each configuration $\left(m_{t},n_{t}\right)$, with $m_{t}\leq n_{t}$

		$m_{t}$								
No. of transactions	Test	1	5	10	20	40	80	100	200	500
$n_{t}=50$	As	0.064	0.039	0.035	0.029	0.026	—	—	—	—
	Ex	0.040	0.017	0.013	0.011	0.010	—	—	—	—
$n_{t}=100$	As	0.083	0.048	0.033	0.023	0.021	0.020	0.019	—	—
	Ex	0.068	0.032	0.019	0.013	0.011	0.010	0.010	—	—
$n_{t}=200$	As	0.102	0.069	0.043	0.025	0.018	0.014	0.016	0.014	—
	Ex	0.095	0.059	0.034	0.018	0.012	0.010	0.011	0.009	—
$n_{t}=500$	As	0.141	0.125	0.087	0.052	0.027	0.016	0.014	0.012	0.012
	Ex	0.137	0.120	0.082	0.047	0.023	0.013	0.012	0.010	0.009

Model **7** holds with $\tau_{t}=0$ for each trader. The nominal test size is α=0.01.

## Enemy Brothers: Power and False Positive Rate

When model **7** holds with $\tau_{t}>0$ for one or more traders, we write $T_{NF}=\left\{t:\tau_{t}=0\right\}$ and $T_{F}=\left\{t:\tau_{t}>0\right\}$ for the sets corresponding to noncheating traders and fraudsters, respectively. Power (P) is defined as the proportion of traders in $T_{F}$ that are correctly identified as potential fraudsters. The false positive rate (FPR) is the proportion of rejections of the null hypothesis **6** that turn out to be wrong, since they refer to traders that belong to $T_{NF}$. Both performance measures play a crucial role when antifraud analysis is put into practice. In our simulations we compare the results under different contaminant distributions ${ϒ}_{k}^{\left(t\right)}\left(d_{1},\ldots ,d_{k}\right)$, with k=2.

Our first contamination instance assumes that the first two digits of $\tau_{t}n_{t}$ transactions from trader $t\in T_{F}$ are generated according to the discrete uniform distribution on {10,…,99}. Therefore,

$$\pi_{2}^{\left(t\right)}\left(d_{1},d_{2}\right)=\left(1-\tau_{t}\right)\Psi_{2}^{\left(m_{t},n_{t}\right)}\left(d_{1},d_{2}\right)+\tau_{t}\frac{1}{90},$$ [12]

for $d_{1}\in \left\{1,\ldots ,9\right\}$ and $d_{2}\in \left\{0,\ldots ,9\right\}$. The uniform distribution provides an unfavorable scenario for fraud detection, since ${ϒ}_{2}^{\left(t\right)}\left(d_{1},d_{2}\right)$ is then close to the NBL marginal probability **2** for most digit pairs $\left(d_{1},d_{2}\right)$. Our second contamination scheme instead concentrates frauds on a specific digit pair, say $\left({\bar{d}}_{1},{\bar{d}}_{2}\right)$, randomly selected from the discrete uniform distribution on {10,…,99}. The contaminated model thus becomes

$$\begin{array}{ccc} \pi_{k}^{\left(t\right)}\left(d_{1},d_{2}\right)=\left(1-\tau_{t}\right)\Psi_{2}^{\left(m_{t},n_{t}\right)}\left(d_{1},d_{2}\right)+\tau_{t}I_{\left\{{\bar{d}}_{1},{\bar{d}}_{2}\right\}}\left(d_{1},d_{2}\right). & & \end{array}$$ [13]

Although this Dirac-type contamination may at first sight appear extreme, our experience with manipulated declarations is that similar patterns may arise rather frequently among the transactions found to be fraudulent, especially when contamination is due to the attempt to circumvent threshold-depending duties, either “ad valorem”—that is, computed as a percentage of the declared value—or fixed. In fact, the attempt to declare quantities below the threshold (or above it, according to the specific regulation) typically produces a bias in the corresponding values similar to that represented by a Dirac-type model. Other instances of contamination are considered in *SI Appendix*, section 4.

We consider the simplified case where $\tau_{t}$ is the same for each $t\in T_{F}$. We take $\tau_{t}=0.2,0.5,0.8$, to represent three increasing levels of individual propensity to fraud. We also define the proportion of fraudsters in the whole market as

$$\varsigma =\frac{card\left(T_{F}\right)}{card\left(T\right)},$$

where $T=T_{NF}⋃T_{F}$ is the set of all traders. We fix ς=0.05,0.1, to investigate the effect of different degrees of fraud diffusion in the market. Our estimates of P and FPR are based on $T^{†}=10,000$ idealized traders, independently generated in each configuration. Nonmanipulated transactions are again simulated with the algorithm described in *SI Appendix*, section 2. We restrict our analysis to the market configurations for which the NBL approximation to $\Psi_{2}^{\left(m_{t},n_{t}\right)}\left(d_{1},d_{2}\right)$ is good, and the empirical test sizes closely match the nominal one, to avoid confounding between power and lack of fit. We give results only for the configurations with $m_{t}=n_{t}$. Pairs where $m_{t}$ is of the same order of magnitude as $n_{t}$ yield qualitatively similar findings and are not reported.

Table 3 shows the estimated values of P and FPR under the uniform contamination model **12** for $V_{\left\{1\right\}}^{\left(t\right)}$, using the asymptotic quantile $\chi_{8,0.99}^{2}$, and for the TS version of the procedure of Barabesi et al. (6). Not surprisingly, the detection rates are low in the case of sporadic contamination ($\tau_{t}=0.2$). It is apparent that no statistical method can be expected to have high power against “well-masked” frauds, unless the number of contaminated transactions becomes relatively large. Indeed, it is clearly seen that P rapidly grows with both $\tau_{t}$ and $n_{t}$, leading to almost sure detection of fraudsters even through the potentially very conservative TS procedure (e.g., when $\tau_{t}=0.8$ and $n_{t}\geq 200$). Both methods thus prove to be able to identify the traders belonging to $T_{F}$ if there is enough information on the contaminant distribution in the available data, also in the unfavorable framework provided by **[12]**. The value of FPR is much higher with $V_{\left\{1\right\}}^{\left(t\right)}$, as expected, except in some instances of low contamination, where the number of hypotheses **6** rejected by TS is very small and the estimate of FPR is overwhelmed by its sampling variability. The choice between $V_{\left\{1\right\}}^{\left(t\right)}$ and TS should then depend on the user’s attitude toward FPR and toward the power reduction implied by TS in situations of intermediate contamination. The value of ς does not have a major impact on P, thus suggesting that our procedures can be equally effective in detecting isolated fraudsters and more diffuse illegal trading behavior. However, a considerable increase in FPR is to be expected in the former situation, especially for $V_{\left\{1\right\}}^{\left(t\right)}$.

**Table 3.** Uniform contamination model 12

		ς=0.05						ς=0.10					
		$\tau_{t}=0.2$		$\tau_{t}=0.5$		$\tau_{t}=0.8$		$\tau_{t}=0.2$		$\tau_{t}=0.5$		$\tau_{t}=0.8$	
Trade configuration	Test	P	FPR	P	FPR	P	FPR	P	FPR	P	FPR	P	FPR
$n_{t}=50$	$V_{\left\{1\right\}}^{\left(t\right)}$	0.034	0.865	0.196	0.546	0.586	0.302	0.030	0.779	0.200	0.346	0.574	0.178
$m_{t}=50$	TS	0.002	0.000	0.008	0.000	0.154	0.013	0.000	1	0.019	0.000	0.133	0.007
$n_{t}=100$	$V_{\left\{1\right\}}^{\left(t\right)}$	0.058	0.788	0.436	0.297	0.938	0.184	0.043	0.705	0.425	0.175	0.924	0.097
$m_{t}=100$	TS	0.004	0.000	0.070	0.054	0.574	0.003	0.002	0.667	0.063	0.000	0.539	0.002
$n_{t}=200$	$V_{\left\{1\right\}}^{\left(t\right)}$	0.060	0.778	0.810	0.179	1	0.151	0.097	0.484	0.801	0.109	1	0.097
$m_{t}=200$	TS	0.006	0.500	0.356	0.000	0.964	0.002	0.005	0.444	0.345	0.003	0.959	0.004
$n_{t}=500$	$V_{\left\{1\right\}}^{\left(t\right)}$	0.272	0.401	1	0.160	1	0.154	0.281	0.226	1	0.069	1	0.081
$m_{t}=500$	TS	0.028	0.263	0.932	0.000	1	0.004	0.029	0.065	0.928	0.000	1	0.000

Shown are estimated power (P) and false positive rate (FPR) for the first-digit statistic $V_{\left\{1\right\}}^{\left(t\right)}$, using the asymptotic quantile $\chi_{8,0.99}^{2}$, and for the TS version of the procedure of Barabesi et al. (6), based on $T^{†}=10,000$ Monte Carlo replicates for each pair $\left(m_{t},n_{t}\right)$. The nominal test size is α=0.01.

Table 4 repeats the analysis under the Dirac-type scheme **13**. The contaminant distribution is now well separated from $\Psi_{2}^{\left(m_{t},n_{t}\right)}\left(d_{1},d_{2}\right)$ and both methods generally have excellent detection properties, with some minor differences only in the problematic case $\tau_{t}=0.2$. However, FPR is much higher for $V_{\left\{1\right\}}^{\left(t\right)}$. In such contamination frameworks the TS procedure thus comes closer to performing like an “ideal” test, leading to the identification of most potential fraudsters with a very small number of false alarms. The effect of ς is still minor on P, while it is more noticeable on FPR for $V_{\left\{1\right\}}^{\left(t\right)}$.

**Table 4.** The same as Table 3, but now for contamination model 13

		ς=0.05						ς=0.10					
		$\tau_{t}=0.2$		$\tau_{t}=0.5$		$\tau_{t}=0.8$		$\tau_{t}=0.2$		$\tau_{t}=0.5$		$\tau_{t}=0.8$	
Trade configuration	Test	P	FPR	P	FPR	P	FPR	P	FPR	P	FPR	P	FPR
$n_{t}=50$	$V_{\left\{1\right\}}^{\left(t\right)}$	0.712	0.218	0.998	0.199	1	0.184	0.696	0.121	0.996	0.092	1	0.108
$m_{t}=50$	TS	0.520	0.763	1	0.002	1	0.002	0.555	0.005	1	0.003	1	0.001
$n_{t}=100$	$V_{\left\{1\right\}}^{\left(t\right)}$	0.876	0.189	1	0.188	1	0.145	0.891	0.095	1	0.083	1	0.081
$m_{t}=100$	TS	0.972	0.008	1	0.004	1	0.000	0.980	0.001	1	0.003	1	0.001
$n_{t}=200$	$V_{\left\{1\right\}}^{\left(t\right)}$	0.972	0.169	1	0.167	1	0.150	0.967	0.091	1	0.079	1	0.076
$m_{t}=200$	TS	1	0.004	1	0.006	1	0.000	1	0.002	1	0.002	1	0.000
$n_{t}=500$	$V_{\left\{1\right\}}^{\left(t\right)}$	1	0.171	1	0.158	1	0.176	1	0.078	1	0.094	1	0.071
$m_{t}=500$	TS	1	0.006	1	0.000	1	0.000	1	0.002	1	0.003	1	0.003

## Corrections to Goodness-of-Fit Statistics

We now focus on the trading configurations for which the NBL does not provide a satisfactory representation of the genuine digit distribution $\Psi_{k}^{\left(m_{t},n_{t}\right)}\left(d_{1},\ldots ,d_{k}\right)$, that is, when $m_{t}\ll n_{t}$. In this case, the reported distributional results are no longer valid for $V_{\left\{1,\ldots ,k\right\}}^{\left(t\right)}$ or for the exact Monte Carlo approach of Barabesi et al. (6). The true probability $\Psi_{k}^{\left(m_{t},n_{t}\right)}\left(d_{1},\ldots ,d_{k}\right)$ should replace the NBL version of $\pi_{k}^{\left(t\right)}\left(d_{1},\ldots ,d_{k}\right)$ in **[9]** to obtain valid tests of hypothesis **6**. Since $\Psi_{k}^{\left(m_{t},n_{t}\right)}\left(d_{1},\ldots ,d_{k}\right)$ is unknown, we resort to our Monte Carlo algorithm for simulating nonfraudulent transactions and we compute a model-free approximation to the null distribution function of $V_{\left\{1,\ldots ,k\right\}}^{\left(t\right)}$. This approximation is then used to obtain a test of **[6]**. Similar testing procedures have proved to be useful in other domains, in the case of correlated observations and other distributional misspecifications (e.g., ref. 29 and the references therein).

If t is the trader of interest, let $t^{*}$ be an idealized noncheating trader such that $t^{*}\;\neq \;t$, while $m_{t^{*}}=m_{t}$ and $n_{t^{*}}=n_{t}$. The set of transactions for trader $t^{*}$ is randomly generated according to the algorithm described in *SI Appendix*, section 2, and the resulting statistical values are collected in vector $x^{\left(t^{*}\right)}$, say. Correspondingly, let $V_{\left\{1,\ldots ,k\right\}}^{\left(t^{*}\right)}$ be the test statistic **9** computed for trader $t^{*}$. Under model **7**, the significant-digit random variables associated to the elements of $x^{\left(t^{*}\right)}$ can be considered as independent copies of those associated to the elements of $X^{\left(t\right)}$, in the absence of data manipulation. We thus estimate the unknown null distribution function $F_{V_{\left\{1,\ldots ,k\right\}}^{\left(t\right)}}$ as a Monte Carlo average over $T^{*}$ replicates of $t^{*}$. This yields

$${\hat{F}}_{V_{\left\{1,\ldots ,k\right\}}^{\left(t\right)}}\left(v\right)=\frac{1}{T^{*}}\overset{T^{*}}{\underset{t^{*}=1}{\sum }}I_{]-\infty ,v]}\left(V_{\left\{1,\ldots ,k\right\}}^{\left(t^{*}\right)}\right),$$ [14]

for $v\in R^{+}$, and

$${\hat{\zeta }}_{\gamma }=\inf\left\{v:{\hat{F}}_{V_{\left\{1,\ldots ,k\right\}}^{\left(t\right)}}\left(v\right)\geq \gamma \right\}$$

for the corresponding estimate of the γ quantile. Therefore, we reject hypothesis **6** at nominal test size α, and we consider trader t a potential fraudster, if

$$v_{\left\{1,\ldots ,k\right\}}^{\left(t\right)}>{\hat{\zeta }}_{1-\alpha },$$ [15]

where $v_{\left\{1,\ldots ,k\right\}}^{\left(t\right)}$ is the observed value of $V_{\left\{1,\ldots ,k\right\}}^{\left(t\right)}$.

Motivated by large-scale applications, Efron (30) describes a related methodology for empirically estimating a null distribution when the standard theoretical model (such as the NBL in the case of digit counts) does not hold. This approach uses the available data to estimate an appropriate version of the distribution of the test statistic under the null hypothesis. However, it is apparent that empirical null estimation is not directly feasible when recast in the framework of models **5** and **7**. One reason is that the method generally requires a known parametric form for the null distribution, whose parameters are then estimated from the available realizations of the test statistic. Even more fundamentally, in our applied context there is no guarantee that the proportion of genuine transactions is large for each trader, that is, that $\tau_{t}$ is small for each t in models **5** and **7**, thus violating a key assumption for empirical null estimation (ref. 30, p. 98).

On the other hand, the proportion of transactions that involve manipulated data and their impact on ${\hat{F}}_{V_{\left\{1,\ldots ,k\right\}}^{\left(t\right)}}$ is arguably small when considering the Cartesian products defined in Eq. **10**. First, both $U_{j}$ and $Q_{j}$ are not trader specific, since they contain all of the transactions in the market for the corresponding good, and the resulting idealized transactions are further aggregated to obtain the required basket of $n_{t}$ transactions on $m_{t}$ products. Second, as already reviewed in the statistical background, an intrinsic robustness property of the NBL specification of our contamination model arises from decomposition **4**, since the product of independent random variables follows the NBL if only one of the factors does, regardless of the other factors (ref. 8, p. 188). We may thus expect a reduction in the contamination effect produced by a manipulated element of $U_{j}$ (respectively, $Q_{j}$), after multiplication by a genuine element of $Q_{j}$ (respectively, $U_{j}$). Third, if the NBL does not hold, the contaminant distribution ${ϒ}_{k}^{\left(t\right)}\left(d_{1},\ldots ,d_{k}\right)$ for a trader t may not be too far from the genuine distribution $\Psi_{t\prime }\left(d_{1},\ldots ,d_{k}\right)$ for some other trader t′ ≠ t, which further reduces the degree of anomaly of the corresponding realizations in the whole market. We thus see our estimate ${\hat{F}}_{V_{\left\{1,\ldots ,k\right\}}^{\left(t\right)}}$ as the outcome of an extended null estimation approach, where $F_{V_{\left\{1,\ldots ,k\right\}}^{\left(t\right)}}$ is estimated by exploiting all of the potential samples that could have been observed given the realized transactions in the market. Since the cardinality of this sample space is very large, we finally resort to Monte Carlo simulation for approximating the extended empirical null.

Table 5 reports the estimated sizes $\hat{\alpha }$ for different values of $n_{t}$ and for $m_{t}=1$, when test **15** is performed at α=0.01 on the same sets of t=1,…,85,500 idealized traders already considered in Table 1, and the Monte Carlo average in **[14]** is computed on $T^{*}=10,000$ independent replicates for each value of $n_{t}$. The analysis for the case $m_{t}=5$ is given in *SI Appendix*, section 5. In all instances, comparison with the estimated sizes of the liberal $\chi_{8}^{2}$ test (copied from Table 1) shows that the improvement provided by our procedure is paramount. The appropriate size is also reached when $n_{t}$ grows, while $m_{t}$ is kept fixed. Therefore, our approach provides a valid test of **[6]** even when the asymptotic framework does not comply with the requirements of Hill’s limit theorem.

**Table 5.** Estimates of test size, P, and FPR using modified procedures 15 and 16, with $T^{*}=10,000$, for different values of $n_{t}$ and for $m_{t}=1$

			Uniform contamination (Eq. **12**)				Dirac-type contamination (Eq. **13**)			
		$\tau_{t}=0$	$\tau_{t}=0.5$		$\tau_{t}=0.8$		$\tau_{t}=0.5$		$\tau_{t}=0.8$	
No. of transactions	Test	$\hat{\alpha }$	P	FPR	P	FPR	P	FPR	P	FPR
$n_{t}=100$	$V_{\left\{1\right\}}^{\left(t\right)}$	0.071	0.414	0.716	0.928	0.600	1	0.579	1	0.572
	Test **15**	0.010	0.000	1	0.000	1	0.850	0.167	1	0.180
	Test **16**	0.011	0.350	0.329	0.864	0.179	0.990	0.161	1	0.144
$n_{t}=200$	$V_{\left\{1\right\}}^{\left(t\right)}$	0.094	0.812	0.683	1	0.630	1	0.648	1	0.634
	Test **15**	0.010	0.000	1	0.000	1	0.878	0.157	1	0.187
	Test **16**	0.012	0.678	0.213	0.934	0.182	0.998	0.153	0.992	0.175
$n_{t}=500$	$V_{\left\{1\right\}}^{\left(t\right)}$	0.132	1	0.719	1	0.714	1	0.708	1	0.717
	Test **15**	0.010	0.004	0.983	0.000	1	0.776	0.173	1	0.154
	Test **16**	0.010	0.894	0.189	0.938	0.149	0.996	0.171	1	0.143

The estimated test sizes for $V_{\left\{1\right\}}^{\left(t\right)}$ are also given as a reference. The nominal test size is α=0.01. The number of independent idealized traders in each market configuration is $T^{†}=85,500$ for procedure **15** and $T^{†}=10,000$ for procedure **16**, P and FPR. ς=0.05 when computing P and FPR

We then compute P and FPR for test **15**, under the uniform contamination model **12** and the Dirac-type contamination scheme **13**, using the same sets of t=1,…,10,000 idealized traders already considered in Tables 3 and 4. For simplicity, we restrict our analysis to ς=0.05 and $\tau_{t}=0.5,0.8$, similar qualitative conclusions being reached in the other cases. The results are again reported in Table 5 and in *SI Appendix*, section 5, for $m_{t}=1$ and $m_{t}=5$, respectively. We see that test **15** can have severe difficulties in discriminating between $T_{F}$ and $T_{NF}$, unless $\Psi_{k}^{\left(m_{t},n_{t}\right)}\left(d_{1},\ldots ,d_{k}\right)$ and ${ϒ}_{k}^{\left(t\right)}\left(d_{1},\ldots ,d_{k}\right)$ are well separated or $\tau_{t}$ is close to one. One reason for the observed loss of power is the large number of goods that are potentially involved in the Monte Carlo estimation process. Indeed, $m_{t^{*}}=m_{t}$ for each idealized trader $t^{*}$ contributing to **[14]**, but the specific goods for which the digit distribution is obtained usually vary from trader to trader. This variability inflates the quantile estimate ${\hat{\zeta }}_{\gamma }$, especially when the ratio $n_{t}/m_{t}$ increases.

We can obtain an improved estimate of the required quantile $\zeta_{\gamma }$ by adopting a refined version of model **7**. In this specification the genuine digit distribution depends not only on $m_{t}$, but also on the specific set of goods, say $G_{t}$, dealt with by trader t. Consequently, we now generate the behavior of $T^{*}$ idealized noncheating traders $t^{*}$ with the constraint that $G_{t^{*}}=G_{t}$. Let ${\tilde{F}}_{V_{\left\{1,\ldots ,k\right\}}^{\left(t\right)}}$ denote the corresponding Monte Carlo estimate of $F_{V_{\left\{1,\ldots ,k\right\}}^{\left(t\right)}}$, computed as in **[14]**. Then,

$${\tilde{\zeta }}_{\gamma }=\inf\left\{v:{\tilde{F}}_{V_{\left\{1,\ldots ,k\right\}}^{\left(t\right)}}\left(v\right)\geq \gamma \right\}$$

and hypothesis **6** is rejected at nominal test size α if

$$v_{\left\{1,\ldots ,k\right\}}^{\left(t\right)}>{\tilde{\zeta }}_{1-\alpha }.$$ [16]

The number of ways in which a basket of $m_{t}$ products can be selected out of G possible goods will be huge in any real-world scenario. Computation of ${\tilde{\zeta }}_{\gamma }$ thus becomes trader specific and cannot be automated before knowing the exact composition of $G_{t}$, differently from ${\hat{\zeta }}_{\gamma }$, which depends only on the pair $\left(m_{t},n_{t}\right)$. Nevertheless, estimation time is still acceptable for routine application of the methodology. For instance, in our experiment computation of ${\tilde{\zeta }}_{\gamma }$ using $T^{*}=10,000$ replicates takes on average less than 0.5 s for a trader t with $n_{t}=200$ and $m_{t}=5$.

The performance of the refined test procedure **16** is displayed in Table 5 (for $m_{t}=1$) and in *SI Appendix*, section 5 (for $m_{t}=5$). All of the estimated sizes are very close to the nominal target α=0.01 and similar to those obtained through **[15]**. Power values are comparable for the three reported tests when the genuine and the contaminant digit distributions are well separated. However, our proposals are still preferred since their FPR is considerably lower than for $V_{\left\{1\right\}}^{\left(t\right)}$. It is in the case of intermediate contamination, as under the uniform model, that the refined estimator ${\tilde{\zeta }}_{\gamma }$ shows much higher efficiency than ${\hat{\zeta }}_{\gamma }$. In this instance rule **16** ensures that the reduction in power with respect to the $\chi_{8}^{2}$ test is minor, while keeping considerably lower values of FPR. We thus conclude that, having the appropriate size and power properties comparable to those of the liberal standard procedure, our modified tests **15** and **16** are recommended whenever the attained levels of FPR can be tolerated in practice.

## Case Studies

**Fig. 1.**
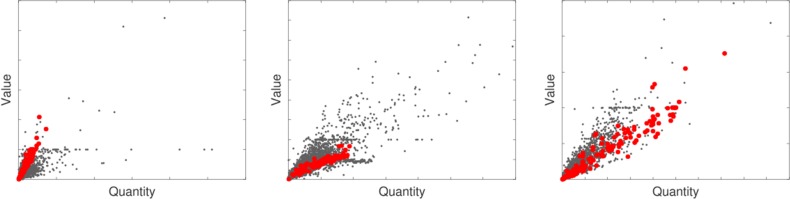
Quantity-value scatter plots for the three most traded products by an Italian operator convicted for two false declarations. The transactions made by this trader are represented as (red) solid circles.

To illustrate the use of the proposed procedure and its ability to detect relevant value manipulations, we first discuss the case of a trader extracted from an archive of fraudulent declarations provided by the Italian customs after appropriate data anonymization. The same archive was also used in ref. 6. The trader under scrutiny has $n_{t}=648$ import transactions on $m_{t}=38$ products from January 2014 to June 2015. The quantities and values appearing in the declarations of the three most traded products (not labeled for confidentiality reasons) are represented as (red) solid circles in the scatter plots of Fig. 1. The information displayed in such scatter plots is the input for some commonly adopted (robust) regression techniques aiming at the automatic detection of value frauds in customs data; see, e.g., ref. 31 and *SI Appendix*, section 7 for further details. However, the plots for this trader do not provide clear evidence of substantial undervaluation or of other major anomalies, although two of the declarations displayed in Fig. 1, *Center* were found to be fraudulent after substantial investigation. Our testing procedure instead produces a strong signal of contamination of the digit distribution. In fact, restricting for simplicity to the first digit, we obtain $v_{\left\{1\right\}}^{\left(t\right)}=62.6$ and ${\hat{\zeta }}_{0.99}=27.3$, based on $T^{*}=10,000$ simulated traders with the same values of $m_{t}$ and $n_{t}$. By applying rule **15**, we can thus conclude that hypothesis **6** can be safely rejected when the focus is shifted from individual transactions, as in Fig. 1, to the whole trader activity, as in our test.

The strength of evidence against the null may suggest the existence in the administrative records of this trader of a larger number of manipulated declarations than the two already detected. It also suggests that our method could be helpful in providing authorities with evidence of potential fraud among traders not previously classified as fraudsters or even not considered as suspicious. In view of contamination models **5** and **7**, and of our simulation results, we expect this information gain to be higher in the case of serial misconduct. Additional investigations for this trader are given in *SI Appendix*, section 6. Although all methods point to the same conclusion, we remark that simple graphical tools for conformance checking—such as histograms—require substantial human interpretation and thus cannot be routinely applied on thousands of traders.

We now move to (anonymized) data provided by the customs office of another EU member state, not disclosed for its specific confidentiality policy, that we label as MS2. The data were collected in the context of a specific operation on undervaluation, focusing on a limited set of products traded by fraudulent operators that have systematically falsified the import values. The traders classified as nonfraudulent were audited by the customs officers of MS2 and no indications of possible manipulation of import values were found. Although the absence of fraud can never be anticipated with certainty, we can thus place good confidence on these statements of genuine behavior. In *SI Appendix*, section 6 and Table S7 we provide empirical investigations of the first-digit distribution of the 15 traders in this small benchmark study for which $n_{t}\geq 50$, as in our simulation experiments. We apply test **16** instead of test **15**, since the available database is limited to a basket of fraud-sensitive products, and we keep α=0.01 and $T^{*}=10,000$ for each observed pair $\left(m_{t},n_{t}\right)$. We give the estimated *P* value of each test, computed as $1-{\tilde{F}}_{V_{\left\{1\right\}}^{\left(t\right)}}\left(v_{\left\{1\right\}}^{\left(t\right)}\right)$, and—as a reference—the asymptotic *P* value from the $\chi_{8}^{2}$ distribution that assumes validity of the NBL. It can be seen that our approach gives very good results, both when applied to fraudsters—it clearly rejects the hypothesis of no contamination for five traders—and in the case of genuine behavior—none of the supposedly honest traders is flagged by our test at α=0.01. Therefore, this study supports the claim that our methodology can be an effective aid to the preparation of the audit plans of customs services, given its ability to point to potential serial fraudsters, in agreement with current guidelines for the customs modernization process (32). We finally note the beneficial effect of our correction for one supposedly honest trader shown in *SI Appendix*, Table S7, whose small basket of traded products may imply spurious deviation from the NBL when the classic $\chi_{8}^{2}$ approximation is used. An extreme example of this effect is also shown in *SI Appendix*, section 6.

## Discussion

We have developed a principled framework for goodness-of-fit testing of the NBL for antifraud purposes, with a focus on customs data collected in international trade. Our approach relies on a trader-specific contamination model, under which fraud detection has close connections with outlier testing. We have given simulation evidence, in the context of a real EU market, showing the features of the traders for which we can expect the genuine digit distribution to be well approximated by the NBL. Our simulation experiment is an empirical study addressing this issue in detail in the context of international trade, where the contrast of fraud has become a crucial task and substantial investigations are often demanding and time consuming. We have also provided simulation-based approximations to the distribution of test statistics when the conditions ensuring the validity of the NBL do not hold. These approximations open the door to the development of goodness-of-fit procedures with good inferential properties and wide applicability.

Our methodology is general and potentially applicable to any country, or year, for which detailed customs data are available. Being mostly automatic, it is suited to be implemented in large-scale monitoring processes in which thousands of traders are screened to find the most suspicious cases. It can also be a valuable aid to the design of efficient and effective audit plans. Although we expect our general guidelines to remain valid in other empirical studies, the specific quantitative findings may clearly vary from one country (year) to another.

A bonus of our contamination approach is that it makes clear the setting in which statistical antifraud analysis takes place. Our conformance testing procedures mainly aim at the detection of serial fraudsters, for which information accumulates in the corresponding transaction records. The generation of low-price clusters of anomalous transactions is a typical consequence of this cheating behavior, and robust clustering techniques can also be used for its detection (e.g., ref. 4). However, rejection of our goodness-of-fit null hypotheses often provides more compelling evidence of fraud, also because it may not be easy to identify the low-price clusters that actually correspond to illegal declarations. Testing conformance to the NBL, or to another suitable distribution for genuine digits, thus shifts the detection focus from individual transactions to the full set of data from each trader.

A word of caution concerns the fact that not all possible frauds can be detected by our method, even when we restrict to manipulation of transaction values. For instance, we cannot expect any statistical procedure (including our own proposal) to have high power against data fabrication methods that preserve the validity of the NBL, at least approximately, and against occasional frauds for which statistical tests are not powerful enough. Therefore, we do not see our methodology as the ultimate antifraud tool, but as a powerful procedure to be possibly coupled with additional information. We support integration of the signals provided by our method with those obtained through alternative statistical techniques and with less technical model-free analyses—such as those developed in refs. 7 and 10—that can be applied on a restricted number of traders. Indeed, we see our approach as a suitable automatic tool for selecting the most interesting cases for additional qualitative and quantitative investigations, while ensuring control of the statistical properties of the adopted tests.

## Supplementary Material

Supplementary File
