# Supplementary material for: Newcomb–Benford law and the detection of frauds in international trade
Source: Proc Natl Acad Sci U S A. 2018 Dec 10;116(1):106–15. doi: 10.1073/pnas.1806617115 (PMC6320519; doi:10.1073/pnas.1806617115)
Supplement: Supplementary File [file pnas.1806617115.sapp.pdf]

Supplementary Information for

The Newcomb-Benford Law and the detection of frauds in international trade

Andrea Cerioli, Lucio Barabesi, Andrea Cerasa, Mario Menegatti and Domenico Perrotta

Andrea Cerioli and Domenico Perrotta.
E-mail: andrea.cerioli@unipr.it; domenico.perrotta@ec.europa.eu

This PDF file includes:

Supplementary text
Figs. S1 to S12
Tables S1 to S7
References for SI reference citations

www.pnas.org/cgi/doi/10.1073/pnas.1806617115

Supporting Information Text

This appendix contains additional theoretical and empirical results, complementing those given in the main manuscript. Specifically,

- we write our contamination model in the space of transactions (§1)
- we describe our algorithm for simulating genuine international trading behavior (§2)
- we examine the performance of our methods under three alternative contamination models (§4)
- we provide additional simulation results, both for our methods and for alternative techniques (§5)
- we provide additional analysis of real data (§6).

Furthermore, in §3 of this appendix we provide a more detailed explanation of the project that gave rise to this work and we describe how to access two databases of simulated transactions (pseudo-data sets) similar to those analyzed in the main manuscript, the structure of these databases and the features of the code that we used for simulation.

In §7 we introduce the web application (called WebARIADNE) that has been developed to assist customs officers and auditors in large-scale screening of traders, by integrating information on their conformance to the NBL with other signals of potential fraud.

1. Contamination model for transactions

We provide the analogue in the transaction space of contamination model [5] of the main manuscript.

Let the positive random variable $X^{(t)}$ represent a transaction value for trader t. A contamination model for this transaction value is defined as

$$F_{X^{(t)}}(x) = (1-\tau_t)H^{(t)}(x) + \tau_t L^{(t)}(x), \qquad [1]$$

where $H^{(t)}$ is the distribution function of $X^{(t)}$ in the absence of fraud and $L^{(t)}$ is the distribution function of $X^{(t)}$ when the transaction is fraudulent.

Let $\pi_k^{(t)}(d_1,\ldots,d_k)$ be defined as in [5] of the main manuscript. If $X^{(t)}$ follows the two-component mixture distribution function $F_{X^{(t)}}(x)$ given in Equation [1] above, it can be seen from the results in §5 of (1) that $\pi_k^{(t)}(d_1,\ldots,d_k)$ can also be obtained as

$$\begin{aligned}\pi_k^{(t)}(d_1,\ldots,d_k) &= \sum_{z\in\mathbb{Z}}\left(F_{X^{(t)}}(10^z(c_{d_1,\ldots,d_k}+10^{-k+1})) - F_{X^{(t)}}(10^z c_{d_1,\ldots,d_k})\right)\\ &= (1-\tau_t)\Psi_k^{(t)}(d_1,\ldots,d_k) + \tau_t\Upsilon_k^{(t)}(d_1,\ldots,d_k), \qquad [2]\end{aligned}$$

where

$$c_{d_1,\ldots,d_k} = \sum_{l=1}^{k} 10^{k-l} d_l.$$

In such a case,

$$\Psi_k^{(t)}(d_1,\ldots,d_k) = \sum_{z\in\mathbb{Z}}\left(H^{(t)}(10^z(c_{d_1,\ldots,d_k}+10^{-k+1})) - H^{(t)}(10^z c_{d_1,\ldots,d_k})\right),$$

and

$$\Upsilon_k^{(t)}(d_1,\ldots,d_k) = \sum_{z\in\mathbb{Z}}\left(L^{(t)}(10^z(c_{d_1,\ldots,d_k}+10^{-k+1})) - L^{(t)}(10^z c_{d_1,\ldots,d_k})\right).$$

Equation [2] above shows how the digit-contamination model [5] defined in the main manuscript arises from contamination of the original transaction value $X^{(t)}$. This relationship can also be helpful in studying how the existence of a hidden correlation structure in transaction data may affect the digit distribution $\pi_k^{(t)}(d_1,\ldots,d_k)$; see (2–4) for recent research on this topic.

2. Simulation of international trade data

We describe the algorithm used for replicating genuine international trading behavior in one specific EU market by picking unit price and traded quantity at random from the data base of Italian customs declarations in a recent calender year. We also provide economic motivation for adopting this algorithm.

Our reference market is made of a set, say $\mathcal{G} = \{g_1,\ldots,g_G\}$, of $G = 5,447$ different goods imported in Italy from non-EU countries and for which at least 50 transactions have been recorded in the year under consideration. These goods account for 6,265,198 trades, corresponding to about 84% of the total number of trades and to almost 97% of the value of the non-EU import market in Italy in the given year. In our simulation study, the cardinality of the full transaction space $\mathcal{X} = \bigcup_{j=1}^{G}\mathcal{X}_j$ is $\mathrm{card}(\mathcal{X}) = \sum_{j=1}^{G} n_j^2 = 77,671,296,438$, where $\mathcal{X}_j$ and n_j are defined as in Equation [10] of the main manuscript. The goods in $\mathcal{G}$ are specified according to their 10-digit code of the Combined Nomenclature, which is the maximum level of accuracy accessible for both imports and exports in the data base of Italian customs declarations. This level of classification is sufficiently detailed to distinguish the products by their material, function and degree of processing.

Given the chosen values of n_t and m_t, the behavior of trader t in the absence of fraud is simulated as follows.

a) Select randomly m_t elements $g_{t,1}, ..., g_{t,m_t}$ from $\mathcal{G}$ without replacement. The selection probability of each element $g_j \in \mathcal{G}$ is proportional to the number of transactions involving g_j in the whole market.

b) Select randomly m_t integers $n_{t,1}, ..., n_{t,m_t}$, such that $n_{t,j} > 0$, for $j = 1, \ldots, m_t$, and $\sum_{j=1}^{m_t} n_{t,j} = n_t$.

c) From the n_j transactions of good $g_{t,j}$ selected at Step a), extract randomly with replacement $n_{t,j}$ unitary prices from set $\mathcal{U}_j$, say $u_{t,j,1}, \ldots, u_{t,j,n_{t,j}}$, and $n_{t,j}$ traded quantities from set $\mathcal{Q}_j$, say $q_{t,j,1}, \ldots, q_{t,j,n_{t,j}}$. According to Equation [4] of the main manuscript, the element-by-element product of the extracted prices and quantities generates a vector $x_j^{(t)} = (x_{j,1}^{(t)}, \ldots, x_{j,n_{t,j}}^{(t)})$ of $n_{t,j}$ fictitious "statistical values" for good $g_{t,j}$, with $x_{t,j,i} = u_{t,j,i}q_{t,j,i}$.

d) Iterate step c) for $j = 1, \ldots, m_t$, to obtain a vector

$$x^{(t)} = (x_1^{(t)}, \ldots, x_{m_t}^{(t)}) \qquad [3]$$

of n_t "statistical values" free from manipulations, i.e. satisfying the assumption that $\tau_t = 0$ in model [7] of the main manuscript. This vector provides the required realization of $X_1^{(t)}, \ldots, X_{n_t}^{(t)}$.

From an economic standpoint, random coupling of data from $\mathcal{U}_j$ and $\mathcal{Q}_j$ involves the implicit assumption that there is no systematic relationship between prices and quantities in transactions related to good j. This assumption is compatible with various market structures, all widely analyzed by economic theory. A clear separation between the determination of quantity and price in each trader's operations may arise when we assume that traders operate on a perfectly competitive market, which implies that traders are all price takers. This means that no single trader can influence the price of the goods bought in each operation and that changes in prices are caused by aggregate market shocks: demand-side shocks, which cause a change in price and quantity in the same direction, and supply-side shocks, which determine a change in the two variables in opposite directions. The interaction between demand-side and supply-side shocks yields no systematic relationship between changes in aggregate quantities and changes in prices, thus implying the absence of systematic relationship for individual transactions under the assumption of perfect competition. Similarly, the relationship between price and quantity in each transaction may be non-systematic even in the absence of perfect competition. Consider the case where both buyers and sellers have some degree of market power. In this case, quantity and price in each operation depend on the relative strength of the operators and on the result of the bargaining process between them. This implies that the same quantity may be bought by the same trader at different prices in different transactions, again determining (approximate) independence between prices and quantities in the set of each trader's transactions.

3. Data, pseudo-data and code description

The European Union has the legal obligation to defend its own budget, which is formed by customs duties for a considerable share. This manuscript is the latest research output of an institutional collaboration between our research group, the Anti-Fraud Office of the European Union (OLAF) and the Customs Offices of selected Member States of the European Union. The original customs declarations were supplied to us, after appropriate anonymization, by the Italian Customs Office and by the Customs Office of the EU Member State labeled as MS2 in the main manuscript. This supply was done under a confidentiality agreement between the Joint Research Centre of the European Commission and the relevant services of the European Union Member States. The customs declarations are documented in the main manuscript and in §2 above, but they are highly sensitive and cannot be freely distributed. Instead, in this appendix we describe how the interested reader can obtain:

A1) Two databases of *simulated transactions* (pseudo-data sets) replicating those for which the Monte Carlo results of this work have been obtained. The first database refers to Tables 1–4 and Equation [15] in Table 5 of the main article, whereas the second refers to the results for test [16] reported in Table 5 of the main article.

A2) The corresponding databases of *simulated test statistics*, used to evaluate the performance of the methodologies and to compute corrections to test statistics.

These databases allow the interested reader to reproduce, up to simulation error, the Monte Carlo findings reported in the main manuscript.

In this appendix we also describe the code used for simulating transactions and for performing our NBL analysis.

A. Pseudo-data description.

Simulated transactions. The two databases of simulated transactions contain 10,000 records for each configuration of trade, and are archived in the following .zip files:

`DPSimValues_10000.zip`: related to Tables 1–4 and Equation [15] of the main manuscript [size = 15GB]

`DPSimValuesMFixed_10000.zip`: related to test [16] in Table 5 of the main manuscript [size < 1GB]

These files are located at the following address

https://athena.jrc.ec.europa.eu/bscw/bscw.cgi/33358

The interested reader can obtain credentials in order to access the databases and download the data by sending a message to the functional mailbox

`JRC-ATHENA-PUB@ec.europa.eu`

with subject *PNAS2018* and the reader's institution in the body (whenever applicable). The credentials will expire after seven days. A bigger pseudo-data set of 50,000 simulated transactions for each configuration of trade (of size 75GB) is also available upon request.

The simulated transactions are provided in .txt files. Each file refers to a specific market configuration, corresponding to a given number of transactions N and a given number of products M, to a specific type of contamination (corresponding to `uniform`, `rndaccum`, `5accum`, `generalizedNB`), amount of contamination (corresponding to `0.2`, `0.5` and `0.8`) and percentage of fraudsters (corresponding to `0.05`, `0.1` and `0.2`). The following correspondences hold between these quantities and those given in the main manuscript:

- $N = n_t$ in Model [7] of the main manuscript
- $M = m_t$ in in Model [7] of the main manuscript
- `uniform` contamination: Equation [12] of the main manuscript
- `rndaccum` contamination: Equation [13] of the main manuscript
- `5accum` contamination: Equation [4] below
- `generalizedNB` contamination: not used in this work (but a potentially interesting parametric contamination through the Generalized Newcomb-Benford distribution)
- `amount of contamination`: τ_t in in Model [7] of the main manuscript
- `percentage of fraudsters`: ζ in Section "Enemy brothers: Power and False Positive Rate" of the main manuscript.

The specific trade and contamination conditions to which the data refer are reported in the file name.

In each .txt file, the rows correspond to the transactions and the columns to the traders. Therefore, a file corresponding to $N = 50$ has 50 rows and 10,000 columns (in the case of 10,000 simulated traders).

Simulated test statistics. The corresponding databases of simulated test statistics contain – for each market configuration, type of contamination, amount of contamination and percentage of fraudsters (see above) – the values of the chi-squared test statistics computed on the simulated data for the first digit, the second digit and the joint distribution of the first-two digits. The file names are

`DPResults_10000.zip`: related to Tables 1–4 and Equation [15] of the main manuscript

`DPResultsMFixed_10000.zip`: related to test [16] in Table 5 of the main manuscript.

Access details are the same as those for the databases of simulated transactions.

The simulated test statistics are provided as binary Matlab files (.mat). The configuration under which each file is obtained is again reported in the file name. The set of configurations is the same as that given for the data base of simulated transactions.

Each file in `DPResults_10000.zip` contains a Matlab structure array, called `out`, with several fields. The following correspondences hold between the fields reported in `out` and those given in the of the main manuscript:

- `chi2`: chi-squared test statistic on the first digit ($V^{(t)}_{\{1\}}$ from Equation [9] of the main manuscript)
- `chi2_2`: chi-squared test statistic on the second digit ($V^{(t)}_{\{2\}}$ from Equation [9] of the main manuscript)
- `chi2_12`: chi-squared test statistic on the first-two digits ($V^{(t)}_{\{1,2\}}$ from Equation [9] of the main manuscript)
- `adj1`: Monte Carlo first-order correction factor for `chi2` (not used in this work)
- `adj2`: Monte Carlo second-order correction factor for `chi2` (not used in this work)
- `adj3`: Monte Carlo correction factor for the 0.99 quantile of `chi2`: see Equation [15] of the main manuscript.
- `cv12` = exact critical values for `chi2_12`, `chi2_1` and `chi2_2` computed using the procedure of (1)
- `fraudster`: dummy variable identifying if trader t is a fraudster
- `size_power_fdr`: estimated size [11] of the main manuscript, Power (P) and False Positive Rate (FPR) for the following test statistics:

- chi2 ($V^{(t)}_{\{1\}}$ from Equation [9] of the main manuscript)
- chi2 after adjustment adj1 (not used in this work)
- chi2 after adjustment adj2 (not used in this work)
- chi2 after adjustment adj3: see Equation [15] of the main manuscript
- chi2 with Benjamini-Hochberg correction (not used, but mentioned in the main manuscript)
- chi2 with Benjamini-Hochberg correction after adjustment adj1 (not used in this work)
- chi2 with Benjamini-Hochberg correction after adjustment adj2 (not used in this work)
- chi2 with Benjamini-Hochberg correction after adjustment adj3 (not used in this work)
- Two-Stage (TS) procedure of (1).

Each file in DPResultsMFixed_10000.zip contains a Matlab structure array, called outstr, with several fields. The following correspondences hold between the fields reported in outstr and those given in the main manuscript:

- size_power_fdr: estimated size [11] of the main manuscript, Power (P) and False Positive Rate (FPR) for the first eight test statistics listed above
- fraudster: dummy variable identifying if trader t is a fraudster
- adj: matrix containing the adjustment parameters estimated for each trader. Each column corresponds to a trader. The first row contains a trader-specific Monte Carlo first-order correction factor for chi2 (not used in this work). The second row contains a trader-specific Monte Carlo second-order correction factor for chi2 (not used in this work); The third row contains our suggested trader-specific Monte Carlo correction factor for the 0.99 quantile of chi2: see Equation [16] of the main manuscript. The fourth row contains a trader-specific Monte Carlo estimate of the appropriate number of degrees of freedom for the second-order correction factor for chi2 (not used in this work).
- chi2resampling: chi2 on the first digit simulated for each trader
- chi2trader: matrix containing the simulated chi-squared test statistics on the first digit required to implement test [16] of the main manuscript for each trader. Each column corresponds to a trader and reports T^* simulated test statistics for the given trader-specific set of goods.

B. Code description and analysis. Our code is written in Matlab, release R2016a. Any hardware configuration running Matlab is sufficient. Special toolboxes are not required, apart from the Statistical toolbox.

Our code consists in two Master Matlab functions for simulating transactions values and in several Matlab specific functions for computing test statistics. Our first Master function is related to Tables 1–4 and Equation [15] of the main manuscript; our second Master function is instead related to test [16] in Table 5 of the main manuscript. The database of customs declarations is given as an argument to these Master functions. The structure containing the database of customs declarations is a binary Matlab file, which was provided to us by the Italian Customs Office under a confidentiality agreement, after appropriate trader and product anonymization that makes impossible to infer the features of individual operators.

Each set of simulated transactions is analyzed as follows:

- Extract three matrices containing the first, the second, and the first-two significant digits, respectively
- Compute the chi-squared test statistics on the extracted digits.

The Monte Carlo quantiles of Equations [15] and [16] of the main manuscript are obtained internally through Matlab specific functions.

Each of the data structures of simulated transactions contained in the files

DPSimValues_10000.zip and DPSimValuesMFixed_10000.zip

can be analyzed in the same way to reproduce the simulation results given in the main manuscript, with the exception of test [16] in Table 5, up to Monte Carlo error. Notice that the estimated test sizes are computed by aggregating the non-cheating traders across all the configurations that have the same number of transactions and traded products.

The outcome of test [16] in Table 5 of the main manuscript can be replicated, up to Monte Carlo error, by using the simulated test statistics reported in field chi2trader within array outstr in the files arichived in DPResultsMFixed_10000.zip. To reproduce these test statistics, the confidential database of customs declarations should instead be available.

4. Alternative contamination models

In this appendix we consider three alternative contamination models. We show that our main qualitative findings remain unaltered regardless of the chosen contamination scheme. Therefore, the contamination scheme does not appear to have a major impact on the relative performance of the different tests, although the specific power values clearly increase with the strength of contamination.

The first model introduces a Dirac-type contamination, where – using the notation of model [13] of the main manuscript – we force $\bar{d}_1 = 5$ in all the contaminated transactions by sampling $(\bar{d}_1, \bar{d}_2)$ from the discrete Uniform distribution on $\{50, \ldots, 59\}$. As a consequence, the model is

$$\pi_2^{(t)}(d_1, d_2) = (1 - \tau_t)\Psi_2^{(m_t, n_t)}(d_1, d_2) + \tau_t I_{\{5, \bar{d}_2\}}(d_1, d_2). \qquad [4]$$

Table S1 shows Monte Carlo estimates of P and FPR under [4]. It is seen that results closely match, with a further increase in power, those given in Table 4 of the main manuscript.

Table S1. Dirac-type contamination model [4]. Estimated Power (P) and False Positive Rate (FPR) for the first-digit statistic $V_{\{1\}}^{(t)}$, using the asymptotic quantile $\chi^2_{8,0.99}$, and for the two-stage (TS) version of the procedure of (1), based on $T^\dagger = 10,000$ Monte Carlo replicates for each pair (m_t, n_t). The nominal test size is $\alpha = 0.01$.

Trade configuration	Test	$\varsigma = 0.05$						$\varsigma = 0.10$					
		$\tau_t = 0.2$		$\tau_t = 0.5$		$\tau_t = 0.8$		$\tau_t = 0.2$		$\tau_t = 0.5$		$\tau_t = 0.8$	
		P	FPR	P	FPR	P	FPR	P	FPR	P	FPR	P	FPR
$n_t = 50$	$V_{\{1\}}^{(t)}$	0.835	0.195	1	0.194	1	0.158	0.862	0.103	1	0.010	1	0.088
$m_t = 50$	TS	0.104	0.037	1	0.000	1	0.004	0.094	0.011	1	0.000	1	0.000
$n_t = 100$	$V_{\{1\}}^{(t)}$	1	0.188	1	0.157	1	0.182	1	0.084	1	0.083	1	0.095
$m_t = 100$	TS	0.712	0.003	1	0.008	1	0.008	0.681	0.001	1	0.000	1	0.003
$n_t = 200$	$V_{\{1\}}^{(t)}$	1	0.174	1	0.167	1	0.153	1	0.088	1	0.092	1	0.092
$m_t = 200$	TS	1	0.006	1	0.004	1	0.002	1	0.001	1	0.002	1	0.001
$n_t = 500$	$V_{\{1\}}^{(t)}$	1	0.152	1	0.186	1	0.171	1	0.080	1	0.088	1	0.096
$m_t = 500$	TS	1	0.000	1	0.000	1	0.002	1	0.000	1	0.001	1	0.003

The second model of this appendix considers a less extreme contamination scheme based on random choice of the first-two digits from the discrete Uniform distribution on $\{10, \ldots, 59\}$. Therefore, it is more similar to the Uniform contamination model [12] of the main manuscript, of which it represents a practically sensible but more specialized variant. We give results for a choice of values of n_t and m_t, $\varsigma = 0.10$ and $\tau_t = 0.5$. Since Uniform contamination is generally unfavorable for anti-fraud analysis, here we are also interested to see how powerful are our modified statistics [15] and [16] of the main manuscript, when m_t is small. Results are provided in Table S2. As expected, our procedures have good performance even in this case of "intermediate" contamination, with detection rates which are in-between those obtained for the Uniform and Dirac-type models.

Table S2. Discrete Uniform distribution on $\{10, \ldots, 59\}$. Estimated Power (P) and False Positive Rate (FPR) for the first-digit statistic $V_{\{1\}}^{(t)}$, using the asymptotic quantile $\chi^2_{8,0.99}$, for the two-stage (TS) version of the procedure of (1), based on $T^\dagger = 10,000$ Monte Carlo replicates for each pair (m_t, n_t), and for procedures [15] and [16] of the main manuscript. The nominal test size is $\alpha = 0.01$.

Trade configuration	Performance measure	$V_{\{1\}}^{(t)}$	TS	Test [15]	Test [16]
$n_t = 100$	P	0.640	0.030	0.010	0.510
$m_t = 1$	FPR	0.496	0.944	0.917	0.136
$n_t = 100$	P	0.520	0.050	0.430	0.420
$m_t = 10$	FPR	0.402	0.286	0.328	0.208
$n_t = 100$	P	0.620	0.010	0.480	0.490
$m_t = 20$	FPR	0.195	0.500	0.111	0.140
$n_t = 100$	P	0.520	0.010	0.490	0.490
$m_t = 100$	FPR	0.212	0.000	0.222	0.197
$n_t = 200$	P	0.980	0.310	0.000	0.860
$m_t = 1$	FPR	0.434	0.659	0.938	0.104
$n_t = 200$	P	0.960	0.190	0.920	0.850
$m_t = 20$	FPR	0.193	0.095	0.071	0.096
$n_t = 200$	P	0.970	0.230	0.960	0.960
$m_t = 40$	FPR	0.134	0.000	0.103	0.059
$n_t = 200$	P	0.950	0.230	0.950	0.950
$m_t = 200$	FPR	0.059	0.042	0.069	0.078

Our final contamination model may be seen as a combination of the previous two schemes. It assumes that fraudsters fabricate the first two-digits with a number from the discrete Uniform distribution on $\{10, \ldots, 19, 50, \ldots, 59\}$. Therefore, we

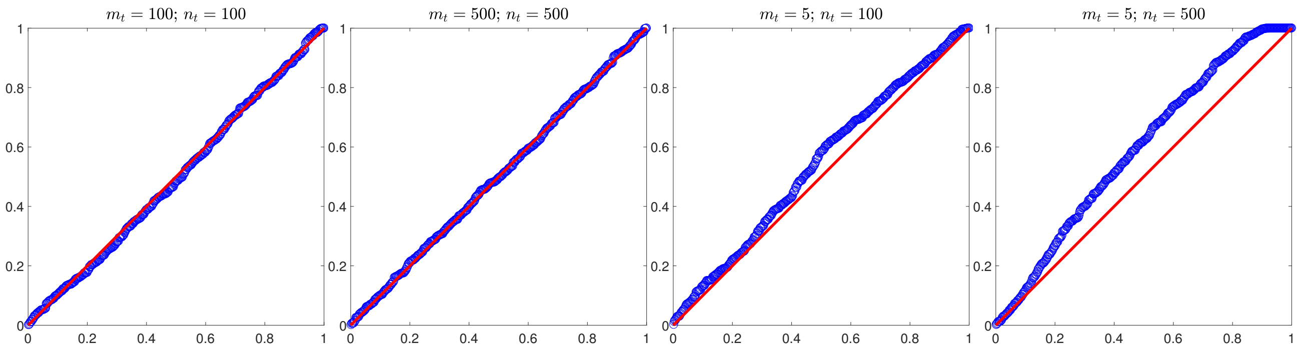

Fig. S1. *Q-Q* plots contrasting the χ^2_8 distribution to the empirical distribution of $V^{(t)}_{\{1\}}$ for samples of 500 "idealized" non-cheating traders under different (m_t, n_t) configurations.

suppose that fraudsters are biased towards choosing the first digit of their transactions from the set $\{1, 5\}$, while the second digit is uniformly distributed. Although only the first digit is restricted to belong to a (small) subset of $\{1, \ldots, 9\}$, we see from Table S3 that performance is close to that reached under the Dirac-type contamination schemes.

Table S3. Discrete Uniform distribution on $\{10, \ldots, 19, 50, \ldots, 59\}$. Estimated Power (P) and False Positive Rate (FPR) for the first-digit statistic $V^{(t)}_{\{1\}}$, using the asymptotic quantile $\chi^2_{8,0.99}$, for the two-stage (TS) version of the procedure of (1), based on $T^\dagger = 10,000$ Monte Carlo replicates for each pair (m_t, n_t), and for procedures [15] and [16] of the main manuscript. The nominal test size is $\alpha = 0.01$.

Trade configuration	Performance measure	$V^{(t)}_{\{1\}}$	TS	Test [15]	Test [16]
$n_t = 100$	P	1.000	0.830	0.000	0.970
$m_t = 1$	FPR	0.408	0.381	1.000	0.067
$n_t = 100$	P	1.000	0.830	1.000	0.980
$m_t = 10$	FPR	0.138	0.057	0.099	0.067
$n_t = 100$	P	1.000	0.820	1.000	1.000
$m_t = 20$	FPR	0.123	0.012	0.029	0.083
$n_t = 100$	P	1.000	0.800	1.000	1.000
$m_t = 100$	FPR	0.083	0.000	0.057	0.065
$n_t = 200$	P	1.000	1.000	0.000	0.990
$m_t = 1$	FPR	0.500	0.438	1.000	0.108
$n_t = 200$	P	1.000	1.000	1.000	1.000
$m_t = 20$	FPR	0.153	0.020	0.091	0.065
$n_t = 200$	P	1.000	1.000	1.000	1.000
$m_t = 40$	FPR	0.138	0.010	0.099	0.091
$n_t = 200$	P	1.000	1.000	1.000	1.000
$m_t = 200$	FPR	0.057	0.000	0.065	0.057

5. Additional simulation results

A. Complements to the main manuscript. We provide additional Monte Carlo results that complement those given in the main manuscript.

A.1. Empirical distribution of the test statistic. We investigate the fit of the whole empirical distribution of $V^{(t)}_{\{1\}}$ to the nominal χ^2_8 distribution in a few selected cases. Figure S1 displays the *Q-Q* plots obtained with four samples of 500 "idealized" non-cheating traders under different (m_t, n_t) configurations. It is apparent that the χ^2_8 approximation is excellent over all the distribution support, not only in the right tail, when $m_t = n_t$. On the other hand, the pictures show the inadequacy of the fit when m_t is of a lower order of magnitude than n_t.

A.2. Monte Carlo results for $m_t = 5$. In Table S4 we report Monte Carlo estimates of test size, P and FPR for our modified procedures [15] and [16] in the case $m_t = 5$.

B. Alternative diagnostic techniques. We provide simulation results for anti-fraud tools based on the fit of the NBL that do not involve the chi-squared statistic [9] of the main manuscript. Therefore, these tools may turn out to be helpful alternatives in situations where chi-squared tests have serious shortcomings (5, Ch. 37).

We start by considering the mean absolute deviation (MAD) criterion of (6, p. 34). In the notation of Equation [9] of the

Table S4. Estimates of test size, P and FPR using modified procedures [15] and [16] of the main manuscript, with $T^* = 10,000$, for different values of n_t and for $m_t = 5$. The estimated test sizes for $V^{(t)}_{\{1\}}$ are also given as a reference. The nominal test size is $\alpha = 0.01$. The number of independent "idealized" traders in each market configuration is $T^\dagger = 85,500$ for procedure [15] and $T^\dagger = 10,000$ for procedure [16], P and FPR. $\varsigma = 0.05$ when computing P and FPR.

Trade configuration	Test		Uniform contamination [12]				Dirac-type contamination [13]			
		$\tau_t = 0$	$\tau_t = 0.5$		$\tau_t = 0.8$		$\tau_t = 0.5$		$\tau_t = 0.8$	
		$\hat{\alpha}$	P	FPR	P	FPR	P	FPR	P	FPR
$n_t = 100$	$V^{(t)}_{\{1\}}$	0.045	0.462	0.653	0.924	0.462	1	0.455	1	0.453
	Test [15]	0.010	0.034	0.828	0.466	0.292	1	0.158	1	0.157
	Test [16]	0.010	0.342	0.308	0.796	0.171	0.992	0.173	0.998	0.166
$n_t = 200$	$V^{(t)}_{\{1\}}$	0.069	0.806	0.622	1	0.561	1	0.569	1	0.570
	Test [15]	0.010	0.022	0.908	0.304	0.348	1	0.169	1	0.169
	Test [16]	0.010	0.592	0.229	0.876	0.189	0.990	0.133	1	0.164
$n_t = 500$	$V^{(t)}_{\{1\}}$	0.126	0.998	0.711	1	0.697	1	0.703	1	0.704
	Test [15]	0.010	0.008	0.970	0.172	0.434	1	0.165	1	0.151
	Test [16]	0.009	0.804	0.191	0.920	0.176	0.998	0.181	0.998	0.170

main manuscript, the first-digit MAD statistic for trader t is

$$\mathrm{MAD}^{(t)}_{\{1\}} = \frac{\sum_{d_1=1}^{9} \left| N^{(t)}_1(d_1) - n_t \rho_1(d_1) \right|}{9}.$$

Since there are no theoretical critical values for $\mathrm{MAD}^{(t)}_{\{1\}}$ (6, p. 158), we have obtained an exact MAD test by using the algorithm of (1). We have also developed modified MAD tests similar to procedures [15] and [16] of the main manuscript, to ensure applicability also when m_t is small. Table S5 reports the size of the tests in the absence of fraud, as well as Power and False Positive Rate in the case of the Uniform contamination model [12] of the main manuscript, for a choice of values of n_t and m_t, $\varsigma = 0.10$ and $\tau_t = 0.5$.

Similarly, Table S6 repeats the analysis for the Z tests, say $\mathrm{Z}^{(t)}_1$ and $\mathrm{Z}^{(t)}_2$, suggested by Kossovsky (5, Ch. 36). These Z tests are performed digit by digit and are designed to give information about which specific digits are responsible for rejection of the null hypothesis. In particular, $\mathrm{Z}^{(t)}_1$ and $\mathrm{Z}^{(t)}_2$ verify the hypotheses that $P(D_1(X^{(t)}) = 1)$ and $P(D_1(X^{(t)}) = 2)$ correspond to the NBL values 0.30103 and 0.17609, respectively. Since the Z tests are based on standardized statistics for which the Central Limit Theorem holds, we have compared the observed values of $\mathrm{Z}^{(t)}_1$ and $\mathrm{Z}^{(t)}_2$ to the asymptotic 0.01 critical value taken form the Standard Normal distribution (see 6, Ch. 6).

The individual Z tests appear to be slightly less liberal than MAD and comparable to the TS approach considered in the main manuscript. However, it should be noted that multiple testing issues arise if $\mathrm{Z}^{(t)}_1$ and $\mathrm{Z}^{(t)}_2$ (and further digit tests) are performed in sequence, so that the overall error rates will become larger than those reported in each column of Table S6. We then conclude that, regardless of the differences in individual conformance measures, the main findings of our work remain unchanged and confirm the importance of the ratio m_t/n_t on the accuracy of the NBL approximation for genuine transactions. This stability also reinforces the idea that our correction approach is very general, being easily adaptable to the specific statistic chosen by the anti-fraud analyst.

Additional anti-fraud tools that have proven to be useful in other domains include the Last-Two digit (LTD) test (5, Ch. 26) and the so-called Digital Development Pattern (DDP) (5, Ch. 33). However, these techniques are not well suited to the context of international trade data that we consider in our work, since they require a considerably larger number of observations than is typically available for a single trader. For instance, for every trader, DDP analyzes the first-digit distributions of the declared values in each interval defined by $\left[10^k, 10^{k+1}\right)$. If we take the availability of more than 100 observations for at least 3 of such intervals as the minimal requirement for application of DDP, only 911 traders would satisfy this condition in the Italian customs archive that we have used for generating genuine transactions in the main manuscript. In addition, the LTD test may be affected by rounding errors – which are not interesting for anti-fraud purposes and which may be due to unknown rounding conventions adopted by customs officers – to a greater extent than $V^{(t)}_{\{1\}}$ and $V^{(t)}_{\{1,2\}}$. Nevertheless, we speculate that our approach might be potentially extended also to these methods in the anti-fraud applications for which they provide suitable tools.

6. Case studies: Additional data analysis

In this appendix we provide further data analysis on the case studies analyzed in the main manuscript. Specifically, we report:

- additional investigations on the Italian trader with fraudulent declarations
- details of empirical results for the benchmark study involving traders from EU Member State MS2
- one synthetic example of the first digit distribution that may occur when m_t is small.

Table S5. Estimated Size (S), Power (P) and False Positive Rate (FPR) for the first-digit statistic $\mathrm{MAD}^{(t)}_{\{1\}}$, using an exact test based on the procedure of (1), and for the MAD-type version of procedures [15] and [16] of the main manuscript. $T^{\dagger} = 10,000$ Monte Carlo replicates for each pair (m_t, n_t). The nominal test size is $\alpha = 0.01$. P and FPR are computed under the Uniform contamination model [12] of the main manuscript.

Trade configuration	Performance measure	$\mathrm{MAD}^{(t)}_{\{1\}}$	MAD-type Test [15]	MAD-type Test [16]
$n_t = 100$	S	0.077	0.010	0.012
$m_t = 1$	P	0.370	0.000	0.300
	FPR	0.651	1.000	0.268
$n_t = 100$	S	0.027	0.011	0.010
$m_t = 10$	P	0.420	0.180	0.330
	FPR	0.364	0.357	0.214
$n_t = 100$	S	0.014	0.007	0.008
$m_t = 20$	P	0.370	0.300	0.290
	FPR	0.260	0.167	0.194
$n_t = 100$	S	0.011	0.018	0.008
$m_t = 100$	P	0.400	0.440	0.400
	FPR	0.200	0.267	0.149
$n_t = 200$	S	0.086	0.016	0.007
$m_t = 1$	P	0.790	0.000	0.640
	FPR	0.494	1.000	0.086
$n_t = 200$	S	0.032	0.012	0.014
$m_t = 20$	P	0.790	0.640	0.690
	FPR	0.269	0.147	0.159
$n_t = 200$	S	0.011	0.002	0.003
$m_t = 40$	P	0.690	0.640	0.650
	FPR	0.127	0.030	0.044
$n_t = 200$	S	0.006	0.013	0.007
$m_t = 200$	P	0.760	0.780	0.740
	FPR	0.062	0.133	0.075

A. Italian fraudster. We focus on the trader extracted from an archive of fraudulent declarations provided by the Italian Customs after appropriate data anonymization. As a complement to the data analysis provided in the main manuscript, Figure S2 shows the distribution of the first significant digits recorded in the 648 transactions made by this trader (blue histogram), together with the theoretical counts expected under the NBL (red line). To provide an empirical reference distribution, the same figure also shows the first significant digit distribution estimated in a set of 10,000 simulated genuine transactions involving the same basket of goods dealt with by this trader (yellow histogram). In this example, where m_t is relatively large, the empirical reference distribution is close to the theoretical NBL values.

Visual inspection of the observed digit distribution confirms this trader as a highly suspicious one. The same conclusion is reached by looking at the alternative statistics (see SI Appendix S.5) $\mathrm{MAD}^{(t)}_{\{1\}} = 0.0243$ and $\mathrm{Z}^{(t)}_1 = 4.84$, both with P-values very close to 0. Different diagnostics thus convey very similar information in this particular case, although graphical tools are not well suited for routine implementation on thousands of traders.

B. Traders from MS2. Table S7 reports detailed empirical results when our approach is applied to fraudulent (F) and non-fraudulent (NF) traders with at least 50 transactions from the benchmark study involving audits made by the Customs Office of the EU Member State labeled as MS2. We recall that the data were collected in the context of a specific operation on undervaluation, focusing on a limited set of products traded by fraudulent operators that have systematically falsified the import values. The traders classified as non-fraudulent were audited by the Customs officers of MS2 and no indications of possible manipulation of import values were found.

Figure S3 and Figure S4 complement the quantitative information in this benchmark study for two fraudsters and two non-fraudulent traders, respectively, by showing the observed distributions of first digits, the theoretical reference distribution under the NBL and the empirical reference distribution obtained by simulating 10,000 genuine transactions from the same traders. Again, it is reassuring to see the good agreement between our clear signals of fraud and visual deviations from the NBL. It is instead difficult to visually judge the relevance of the observed spikes for traders NF1 and NF2. We then conclude that quantitative information is clearly preferable in the case of these two non-fraudulent traders.

The computation of MAD and Z tests essentially replicates the findings already given in Table S7 above. We thus omit the results. However, we note that multiple testing issues may arise when performing Z tests in sequence to see which specific digit is responsible for rejection of the first-order NBL.

C. A trader with small variability. We conclude our empirical analysis by showing the dangerous effects of limited variability of digit values when conformance to NBL is examined. This situation typically occurs if the value of m_t is small and the corresponding basket of traded products only shows a reduced number of possible prices and/or quantities, due to product/market specific reasons or to the limited number of transaction for such products.

Table S6. As Table S5, but now for the Z tests of (5, Ch. 36).

Trade configuration	Performance measure	$Z_1^{(t)}$	Z_1-type Test [15]	Z_1-type Test [16]	$Z_2^{(t)}$	Z_2-type Test [15]	Z_2-type Test [16]
$n_t = 100$	S	0.044	0.009	0.008	0.034	0.003	0.006
$m_t = 1$	P	0.310	0.000	0.220	0.050	0.000	0.010
	FPR	0.563	1.000	0.241	0.861	1.000	0.833
$n_t = 100$	S	0.010	0.001	0.002	0.020	0.003	0.007
$m_t = 10$	P	0.190	0.040	0.170	0.010	0.000	0.000
	FPR	0.321	0.200	0.105	0.947	1.000	1.000
$n_t = 100$	S	0.009	0.004	0.003	0.017	0.004	0.008
$m_t = 20$	P	0.210	0.180	0.180	0.010	0.000	0.010
	FPR	0.276	0.182	0.143	0.938	1.000	0.875
$n_t = 100$	S	0.007	0.010	0.006	0.010	0.004	0.004
$m_t = 100$	P	0.250	0.340	0.230	0.000	0.000	0.000
	FPR	0.194	0.209	0.179	1.000	1.000	1.000
$n_t = 200$	S	0.071	0.011	0.003	0.062	0.004	0.004
$m_t = 1$	P	0.620	0.000	0.500	0.120	0.000	0.050
	FPR	0.508	1.000	0.057	0.824	1.000	0.444
$n_t = 200$	S	0.016	0.006	0.007	0.014	0.001	0.003
$m_t = 20$	P	0.600	0.380	0.500	0.030	0.000	0.020
	FPR	0.189	0.116	0.107	0.813	1.000	0.600
$n_t = 200$	S	0.014	0.004	0.004	0.010	0.002	0.006
$m_t = 40$	P	0.610	0.460	0.500	0.030	0.030	0.040
	FPR	0.176	0.080	0.074	0.750	0.400	0.556
$n_t = 200$	S	0.002	0.002	0.002	0.009	0.006	0.004
$m_t = 200$	P	0.640	0.640	0.620	0.050	0.030	0.040
	FPR	0.030	0.030	0.031	0.615	0.625	0.500

The trader that we now analyze has $n_t = 558$ transactions on $m_t = 6$ different products. The observed value of the (first-digit) chi-square test is $v_{\{1\}}^{(t)} = 79.17$, which yields an asymptotic P-value very close to 0. Instead, the P-value from our estimate $\widetilde{F}_{V_{\{1,\dots,k\}}^{(t)}}(v)$ of the empirical distribution of the test statistic (see [16] in the main manuscript) is 0.217. The reason of the discrepancy between the standard NBL analysis and our approach in shown in Figure S5. There, we display the distribution of the first significant digits recorded in the transactions made by this trader (blue histogram), the theoretical counts expected under the NBL (red line) and the empirical reference distribution obtained in a set of 10,000 simulated genuine transactions involving the same basket of goods dealt with by this trader (yellow histogram). It is clearly seen that the variability in the values of the first digit implied by this specific basket of six goods is too small to allow conformance to the NBL. Therefore, applying standard anti-fraud tools, such as the uncorrected chi-squared test or the blue histogram in Figure S5, is very likely to lead to a false discovery in this particular case.

7. WebARIADNE: an EU application for the detection of statistical anomalies and underlying structures in large scale data

Relevant legal issues related to customs valuation are established in (7). The current guidelines of the World Customs Organization (8) on the fight against fraud call for a modernization of the national anti-fraud services and recommends the adoption of tools based on state-of-the-art mathematical, statistical and computer science methods. In the European Union (EU), where the mandate to counter fraud is rooted in its founding Treaties (9, Articles 310 and 325), the Joint Research Centre (JRC) of the European Commission delivers such tools to the law-enforcement partners in the EU Institutions and Member States since decades (the roots of the activity can be dated back to 1995). The role assigned to the JRC in this policy domain comprises the modeling of fraud in pertinent statistical data, the development of the related statistical methods for fraud detection, their product software implementation, their deployment as services accessible to customers, and the routine dissemination of alerts (fraud relevant signals) to authorized users through the web. More precisely, the users access alerts related to trade-based illicit activities through the THESEUS resource, or generate them in full autonomy, on data of their choice, using tools accessible through the web application WebARIADNE. Figure S6 shows the WebARIADNE login page. This appendix illustrates with some figures the WebARIADNE module implementing the NBL approach discussed in this work.

The frame in the left panel of Figure S7 allows the user to select a data set to analyze and the statistical technique to apply. The current choice includes, in addition to our NBL approach (*BENFORD*), robust methods for detecting outliers in regression data such as those displayed in Figure 1 of the main manuscript (*OUTLIERS*), robust methods for detecting outliers in time series (*FSPIKES*) and association analysis for relating anti-fraud signals to relevant external information (*ISXY*). On the right panel of Figure S7 the user has selected a local dataset and is presented with a preview of its content, in order to help the import operation. The user might also want to analyze a previously uploaded data set: Figure S8 shows the preview given when the data set is selected, with the list of the fields and a sample of records.

Once the user has analyzed a data set, the results are stored in a data base linked to WebARIADNE. An arbitrary number

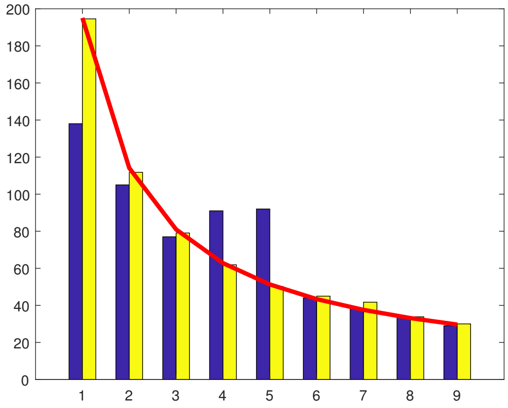

Fig. S2. Italian fraudster: histograms of the distribution of the first significant digits in the 648 transactions made by this trader (blue) and in 10,000 simulated transactions involving the same basket of goods dealt with by this trader (yellow). The red line connects the expected counts under the NBL.

Table S7. Empirical results of a small benchmark study on fraudulent (F) and non-fraudulent (NF) operators from EU Member State MS2, using test [16] with $\alpha = 0.01$ and $T^* = 10,000$ for each pair (m_t, n_t).

Trader	n_t	m_t	$v^{(t)}_{\{1\}}$	P-value from $\widetilde{F}_{V^{(t)}_{\{1,\dots,k\}}}(v)$ (see [16])	P-value from χ^2_8
F1	2991	45	779.2	0.000	0.000
F2	74	6	64.0	0.000	0.000
F3	470	23	109.4	0.000	0.000
F4	80	8	388.8	0.000	0.000
F5	68	9	48.6	0.000	0.000
F6	60	19	16.9	0.033	0.031
F7	204	18	9.88	0.274	0.274
NF1	91	13	10.1	0.264	0.260
NF2	50	18	8.39	0.396	0.396
NF3	62	6	8.22	0.408	0.412
NF4	66	3	6.09	0.642	0.638
NF5	664	4	16.3	0.044	0.038
NF6	62	4	19.6	0.037	0.012
NF7	704	18	8.90	0.381	0.355
NF8	103	29	8.58	0.366	0.379

of result sets can be stored. Therefore, the user is also provided with the possibility to retrieve the results of previous runs. Figure S9 shows an example of the "View Results" frame with five sets of results from *BENFORD* application. The set of results of interest is chosen by clicking on the "I" icon on the right side of the list.

The top panel of Figure S10 shows the results for the five top ranked traders, here with anonymous identifiers. The severity of the trader – which depends on the P-value computed from 10,000 replicates of test [15] of the main manuscript – is shown on the right part of the frame as a colored integer scaled in a range going from 1 to 10. The red asterisk refers to the significance, at $\alpha = 0.01$, of the two-stage (TS) version of the procedure of (1) described in the main manuscript. The two numbers on the left of the severity index indicate the number of declarations and products for that trader, respectively.

Relevant information for each product traded by the selected importer is presented to the user as in the bottom panel of Figure S10. This information includes the market share of the trader, an estimate of the import price applied by the trader, an estimate of the market price for the given product, and an estimate of the deviation of the trader's price from the market price. Figure S11 shows the scatter plot of the imported values and quantities associated to the top listed product – imported 30 times – in the bottom panel of Figure S10; see also Figure 1 in the main manuscript for a different example. It is remarkable to see that there is a rather clear undervaluation associated to the data manipulation detected by the NBL procedure for this trader.

The scatter plot of Figure S12, linked to a NBL signal for another trader and product, instead shows only a mild potential undervaluation, which may remain undetected by the use of an outlier detection method for regression data. Therefore, our NBL test plays a key role in the identification of this potential fraudulent case, which may not be primarily related to underpricing and customs duties evasion[*].

These few examples and considerations suggest that the success of WebARIADNE in orienting control and audit depends on its capacity to combine indicators providing information on different aspects of the fraudulent behavior. However, the potential

[*] Sometimes a mild undervaluation giving rise to a small evasion of import duties is associated to a much larger evasion of VAT in another Member State, obtained by (mis)using the so called Customs procedure 42. In other cases the price level is not relevant at all, because the purpose is to import a different type of product or hide the real country of import.

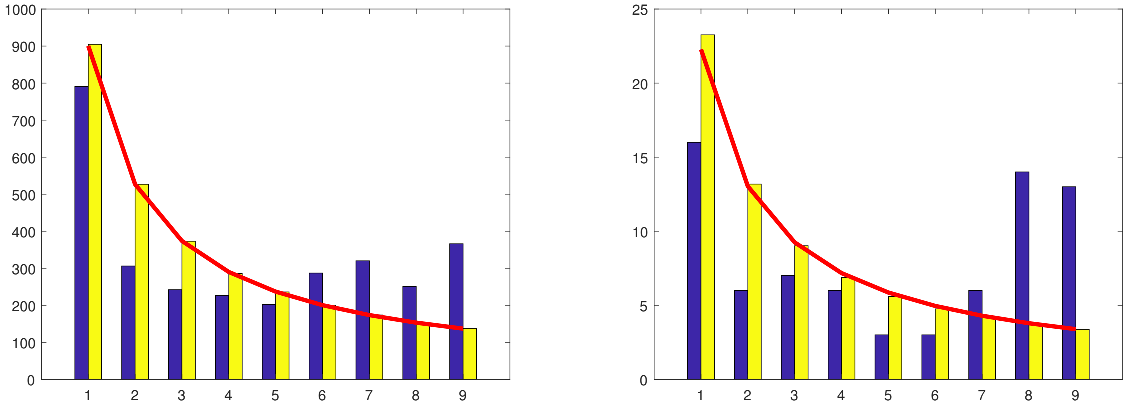

Fig. S3. Traders from MS2: F1 (left) and F2 (right). Histograms of the distribution of the first significant digits in the transactions made by the trader (blue) and in 10,000 simulated transactions involving the same basket of goods dealt with by the trader (yellow). The red line connects the expected counts under the NBL.

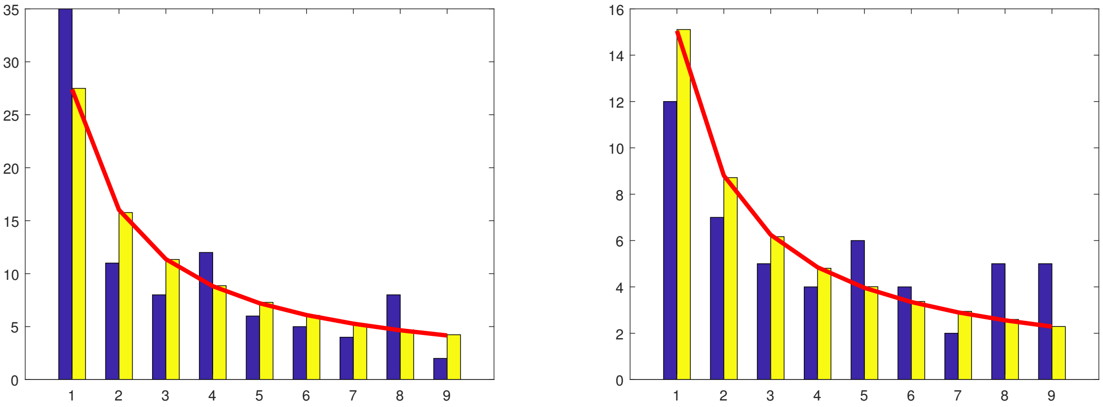

Fig. S4. Traders from MS2: NF1 (left) and NF2 (right). Histograms of the distribution of the first significant digits in the transactions made by the trader (blue) and in 10,000 simulated transactions involving the same basket of goods dealt with by the trader (yellow). The red line connects the expected counts under the NBL.

presence of data manipulation revealed by our NBL approach can be seen as a boosting component of the fraud risk level associated to a trader, because it does not depend on specific fraud control problems and, thus, reduces the unpreventable bias introduced by the analyst.

References

1. Barabesi L, Cerasa A, Cerioli A, Perrotta D (2018) Goodness-of-fit testing for the Newcomb-Benford law with application to the detection of customs fraud. *Journal of Business and Economic Statistics* 36:346–358.
2. Durst R, Miller S (2017) Benford's law beyond independence: tracking benford behavior in copula models, (arXiv), Technical Report 1801.00212v1.
3. Becker T, et al. (2018) Benford's law and continuous dependent random variables. *Annals of Physics* 388:350–381.
4. Chenavier N, Massé B, Schneider D (2018) Products of random variables and the first digit phenomenon. *Stochastic Processes and their Applications* 128:1615–1634.
5. Kossovsky AE (2015) *Benford's Law: Theory, The General Law Of Relative Quantities, And Forensic Fraud Detection Applications*. (World Scientific, Singapore).
6. Nigrini MJ (2012) *Benford's Law*. (Wiley, Hoboken).
7. World Trade Organization (WTO) (1994) General Agreement on Tariffs and Trade (EUR-Lex, http://eur-lex.europa.eu).
8. World Customs Organization (2017) Message from the WCO Secretary General (URL: http://www.wcoomd.org/en/media/newsroom/2017/january/message-of-the-wco-secretary-general-on-international-customs-day-2017.aspx).
9. TFEU (2012) Treaty on the functioning of the european union (consolidated version 2012) (EUR-Lex, http://eur-lex.europa.eu/en/treaties/index.htm). Official Journal of the European Union, C 326, 26.10.2012.

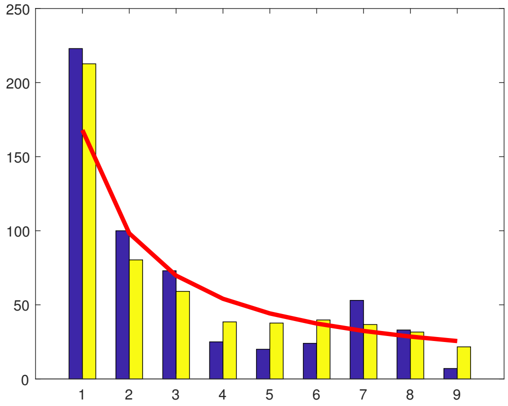

Fig. S5. Trader with $m_t = 6$: histograms of the distribution of the first significant digits in the 558 transactions made by this trader (blue) and in 10,000 simulated transactions involving the same basket of goods dealt with by this trader (yellow). The red line connects the expected counts under the NBL.

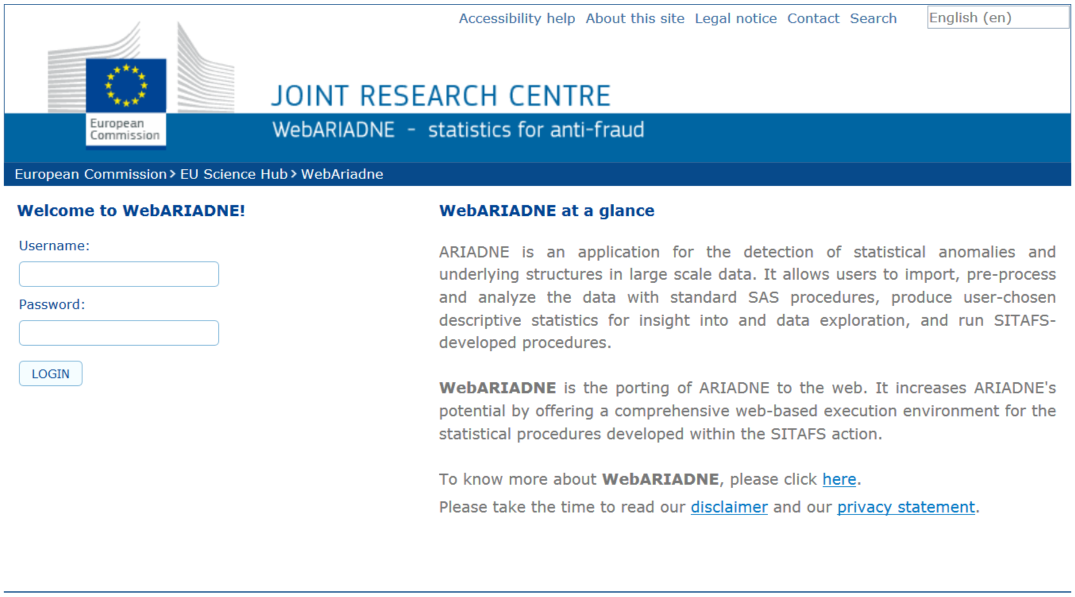

Fig. S6. The login page of the WebARIADNE application, from https://webariadne.jrc.ec.europa.eu.

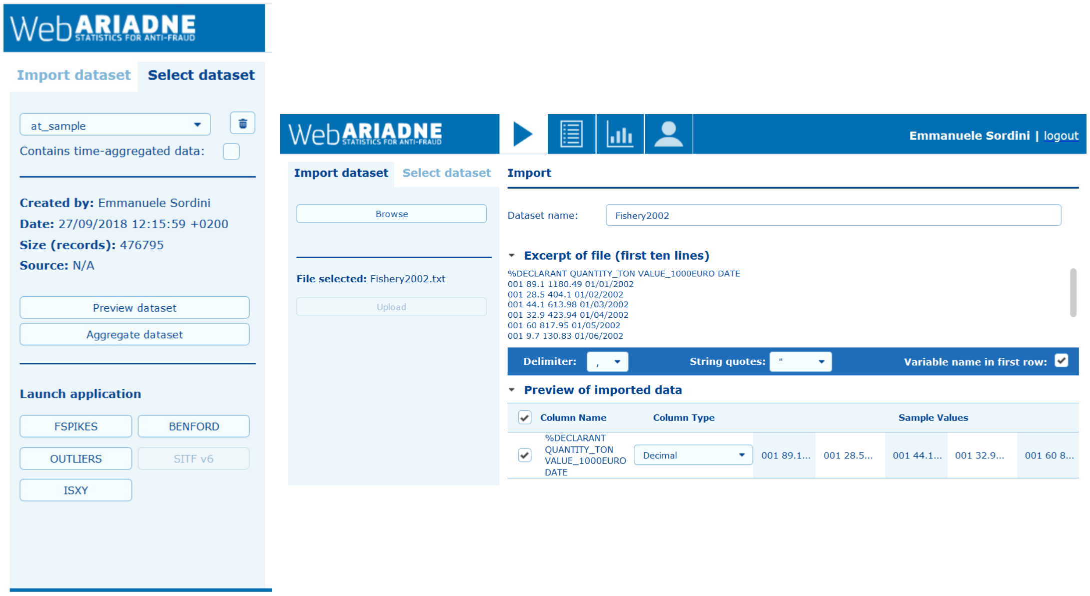

Fig. S7. WebARIADNE application. Left panel: selection of data set and statistical application of interest. Right panel: wizard for importing a new data set (example).

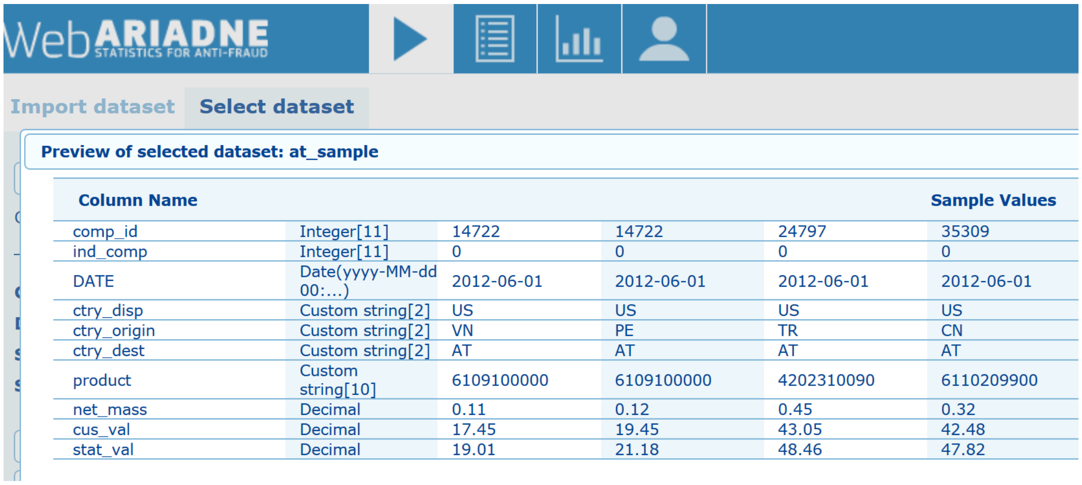

Fig. S8. WebARIADNE application. Selection of an existing data set: example of data preview.

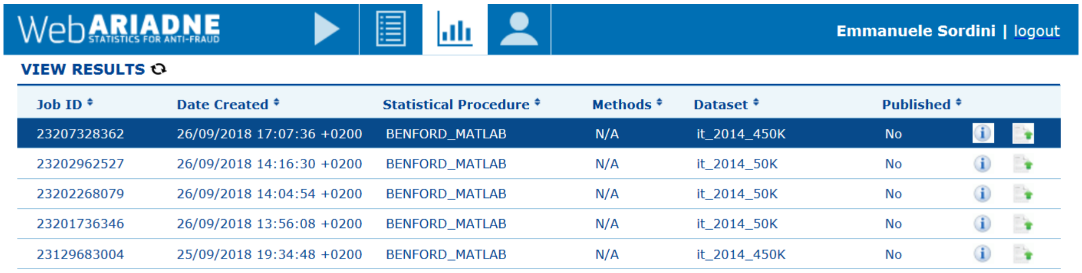

Fig. S9. WebARIADNE application. Selection of an existing set of results obtained with any statistical procedure available in WebARIADNE. The picture shows five sets, all obtained with the *BENFORD* application.

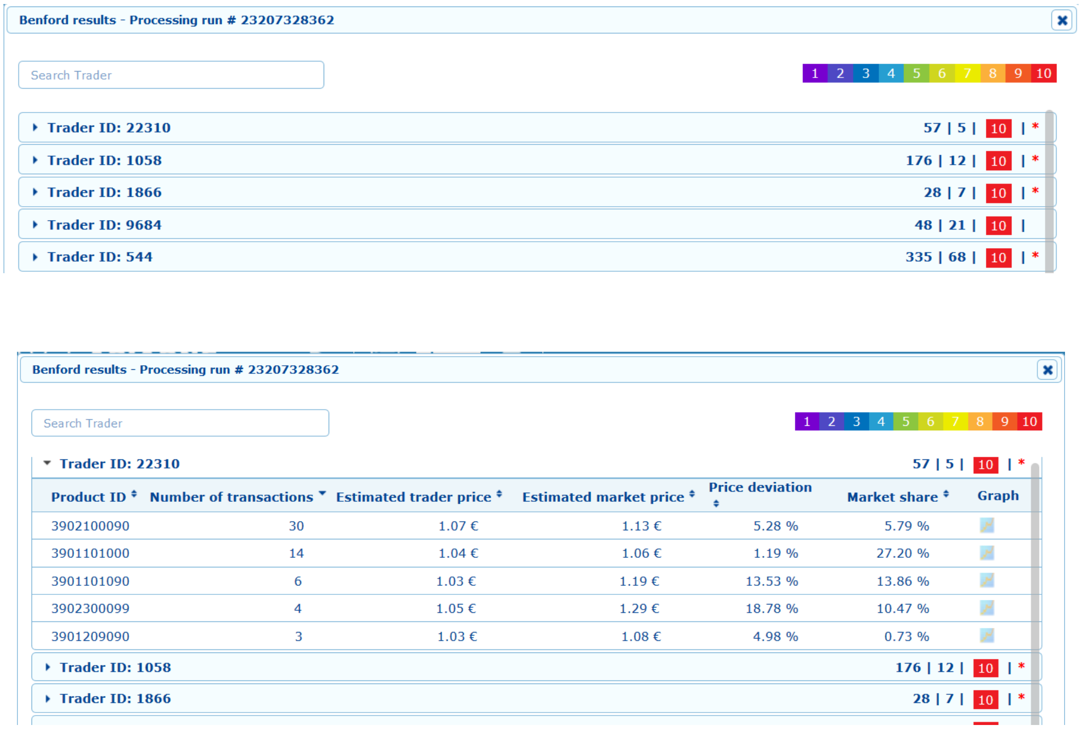

Fig. S10. WebARIADNE application. Results from *BENFORD* application obtained on a subset of the Italian data. Top panel: list of suspected traders. Bottom panel: breakdown of products imported by the trader at the top of the list.

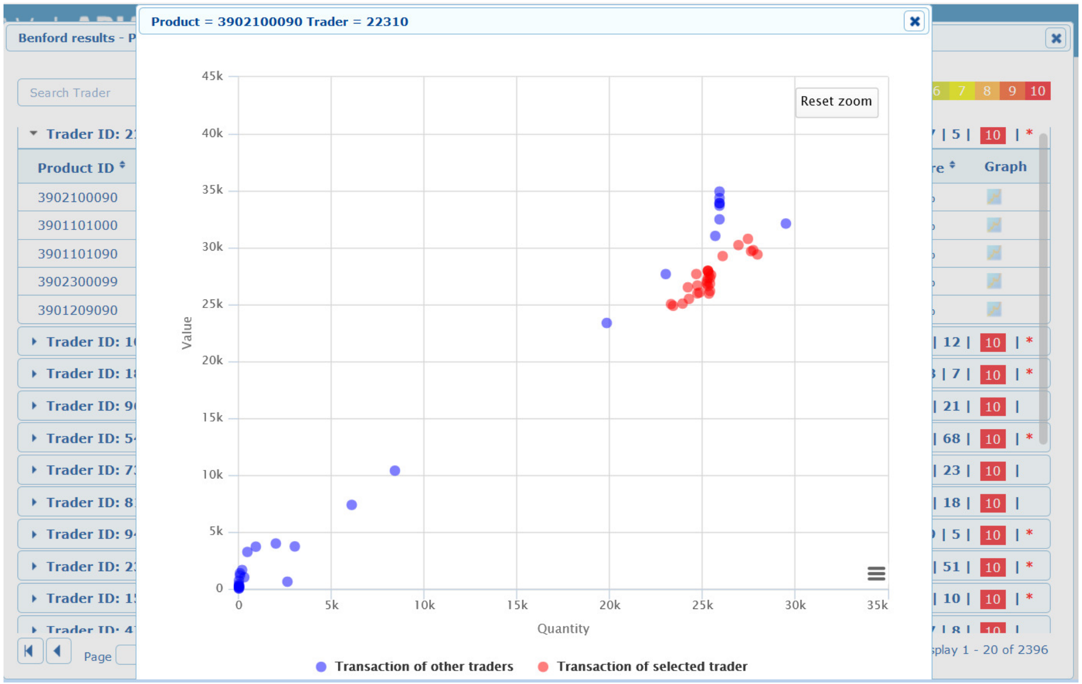

Fig. S11. WebARIADNE application. Scatter plot of the imported values and quantities for the selected trader and product. There is a rather clear undervaluation associated to the data manipulation detected by our NBL procedure.

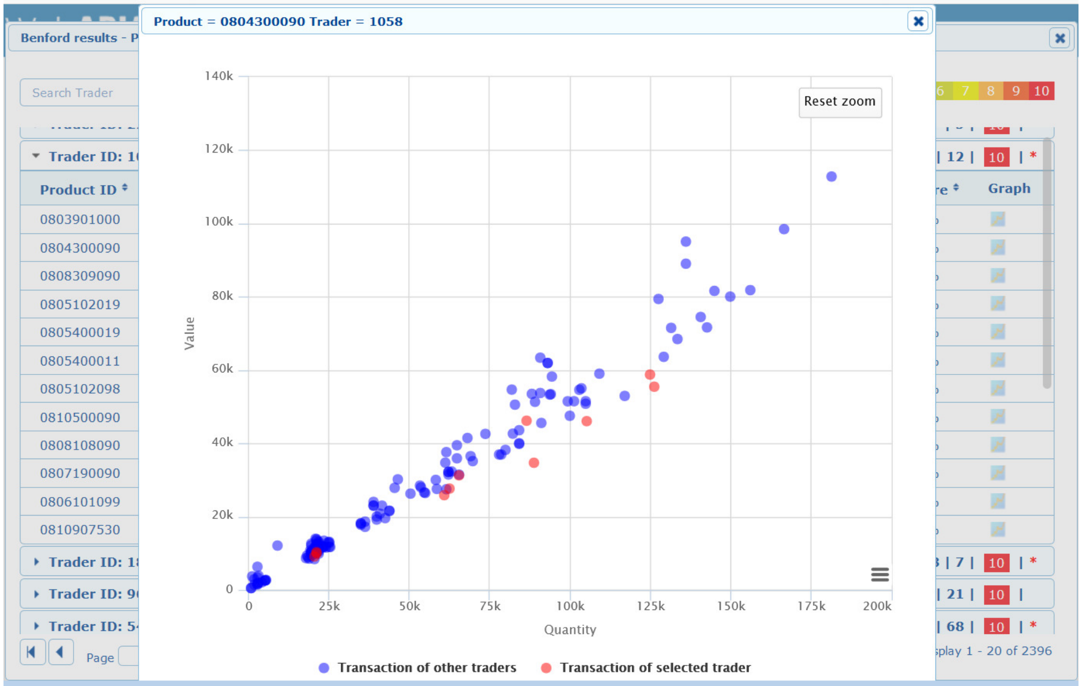

Fig. S12. WebARIADNE application. Scatter plot associated to a second example of signal from *BENFORD* application, for a different trader and product: the association to undervaluation is weaker than the one in Figure S11 and may remain undetected using an outlier detection method for regression data.
